# Luminescent Citrate-Functionalized Terbium-Substituted Carbonated Apatite Nanomaterials: Structural Aspects, Sensitized Luminescence, Cytocompatibility, and Cell Uptake Imaging

**DOI:** 10.3390/nano12081257

**Published:** 2022-04-07

**Authors:** Jaime Gómez-Morales, Raquel Fernández-Penas, Francisco Javier Acebedo-Martínez, Ismael Romero-Castillo, Cristóbal Verdugo-Escamilla, Duane Choquesillo-Lazarte, Lorenzo Degli Esposti, Yaiza Jiménez-Martínez, Jorge Fernando Fernández-Sánchez, Michele Iafisco, Houria Boulaiz

**Affiliations:** 1 Laboratorio de Estudios Cristalográficos, IACT-CSIC-UGR, Avda. Las Palmeras No. 4, 18100 Armilla, Spain; raquel@lec.csic.es (R.F.-P.); j.acebedo@csic.es (F.J.A.-M.); ismaelrc92@gmail.com (I.R.-C.); cristobal.verdugo@csic.es (C.V.-E.); duane.choquesillo@csic.es (D.C.-L.); 2 Institute of Science and Technology for Ceramics (ISTEC), National Research Council (CNR), Via Granarolo 64, 48018 Faenza, Italy; lorenzo.degliesposti@istec.cnr.it (L.D.E.); michele.iafisco@istec.cnr.it (M.I.); 3 Instituto de Biopatología y Medicina Regenerativa (IBIMER), Universidad de Granada, 18016 Granada, Spain; yaijmartinez@correo.ugr.es (Y.J.-M.); hboulaiz@ugr.es (H.B.); 4 Department of Analytical Chemistry, Faculty of Sciences, University of Granada, Avda. Fuentenueva s/n, 18071 Granada, Spain; jffernan@ugr.es

**Keywords:** carbonated-apatites, terbium-doping, citrate-functionalized, luminescence properties, cytocompatibility, intracellular uptake imaging

## Abstract

This work explores the preparation of luminescent and biomimetic Tb^3+^-doped citrate-functionalized carbonated apatite nanoparticles. These nanoparticles were synthesized employing a citrate-based thermal decomplexing precipitation method, testing a nominal Tb^3+^ doping concentration between 0.001 M to 0.020 M, and a maturation time from 4 h to 7 days. This approach allowed to prepare apatite nanoparticles as a single hydroxyapatite phase when the used Tb^3+^ concentrations were (i) ≤ 0.005 M at all maturation times or (ii) = 0.010 M with 4 h of maturation. At higher Tb^3+^ concentrations, amorphous TbPO_4_·nH_2_O formed at short maturation times, while materials consisting of a mixture of carbonated apatite prisms, TbPO_4_·H_2_O (rhabdophane) nanocrystals, and an amorphous phase formed at longer times. The Tb^3+^ content of the samples reached a maximum of 21.71 wt%. The relative luminescence intensity revealed an almost linear dependence with Tb^3+^ up to a maximum of 850 units. Neither pH, nor ionic strength, nor temperature significantly affected the luminescence properties. All precipitates were cytocompatible against A375, MCF7, and HeLa carcinogenic cells, and also against healthy fibroblast cells. Moreover, the luminescence properties of these nanoparticles allowed to visualize their intracellular cytoplasmic uptake at 12 h of treatment through flow cytometry and fluorescence confocal microscopy (green fluorescence) when incubated with A375 cells. This demonstrates for the first time the potential of these materials as nanophosphors for living cell imaging compatible with flow cytometry and fluorescence confocal microscopy without the need to introduce an additional fluorescence dye. Overall, our results demonstrated that Tb^3+^-doped citrate-functionalized apatite nanoparticles are excellent candidates for bioimaging applications.

## 1. Introduction

Luminescent nanoparticles are attractive materials with great potential for nanomedicine as they allow to track their fate in vivo when used as drug carriers, or for applications related to tissue engineering to provide the fluorescent contrast needed to visualize tissues, cells, and tumors [1]. Several luminescent nanomaterials have been investigated for these applications [2,3,4,5,6,7,8,9,10]. Among them, lanthanide-doped calcium phosphates (in particular of the apatite phase) have shown to be promising nanoprobe alternatives to organic fluorophores and quantum dots, as they exhibit different fluorescence emission colors depending on the dopant lanthanide ion, have long luminescence lifetimes, and have an excellent resistance to photobleaching [7,8,9,10,11]. Moreover, different from organic dyes and quantum dots, the main limitations of which are sensitivity to photobleaching [5] and high toxicity levels [12], respectively, apatites are cytocompatible, bioactive, and biodegradable [13]. Apatites also have good stability at physiological conditions, a high capacity to load biomolecules and drugs, and are able to release their payload in response to local stimuli, such as acidic pHs, such as those found at tumor sites or within lysosomes. All these properties have been extensively exploited for preparing apatite-based materials for hard tissue regeneration, as nanocarriers for gene therapy, as drug delivery nanocarriers for targeted treatment of tumor cells, etc. [5,14,15,16,17,18].

To maximize apatite biocompatibility, it is necessary to synthesize biomimetic nanocrystalline apatites [19], which means to produce particles with dimensions, morphologies, and (nano)structural and chemical characteristics that are similar to biological ones. Among the several methods to prepare biomimetic apatites [20], we developed thermal decomplexing of Ca/citrate/phosphate/carbonate [21,22,23,24,25], a wet precipitation method that yields monodisperse, plate-shaped, citrate coated, and carbonated apatite nanoparticles. These nanoparticles have an amount of adsorbed citrate that is similar to that measured in bone apatites [26]. Citrate ions have multiple roles in this precipitation reaction, contributing to the tailoring of the physico-chemical properties of the nanocrystals. In particular, it was demonstrated that citrates favor a flat morphology as a control of particle thickness [24,27] and affect particle interfacial properties by decreasing surface hydrophilicity [28]. The precipitation of carbonated apatite in presence of citrate has also attracted attention for modeling bone mineralization, since this small molecule represents 5.5 wt% of total bone organic matrix [26]. Overall, this methodology has demonstrated to be an effective route to obtain biomimetic carbonated apatites [21], as well as apatites doped with transition metal ions [29], Eu^3+^ [30,31], and even Ca^2+^-doped lanthanide phosphate (LnPO_4_·*n*H_2_O, Ln^3+^ = Eu^3+^ and Tb^3+^, *n*~1) nanophosphors [32,33].

Tb^3+^ has been used as a luminescence activator in different host structures using an excitation wavelength from 300 to 380 nm. Its emission spectra, based on ^5^D_3_→^7^F_j_ (blue) and ^5^D_4_→^7^F_j_ (green) transitions [34], is comparable to Eu^3+^ emission, which possesses a mostly red luminescence. Thanks to its green luminescence, Tb^3+^ doping can be a good alternative to fluorescein-5-isothiocyanate (FITC) and other common fluorescent organic dyes used for nanoparticle surface labeling. Indeed, the use of FITC-labeled apatites and other FITC-labeled nanoparticles poses the risk of FITC detachment in vivo or in vitro [6], leading to misinterpretation in quantifying the nanoparticle signal. Furthermore, surface labeling modifies particle interactions with the surrounding environment, which can impact, for example, the formation of protein corona [35]. All these issues can be avoided by using an internal label, such as Tb^3+^ doping. Fluorescence labeling is necessary when using flow cytometry and confocal fluorescence microscopy to quantify and image nanoparticle uptake into cells [35,36]. The use of Tb^3+^-doped citrate-functionalized carbonated apatite (Tb^3+^:cit-cAp) nanoparticles can therefore be a good alternative to surface labeled apatites, preserving, in addition, the biomimetic surface properties of non-doped nanoparticles. In addition to the above features, Tb^3+^ has been found to have bactericidal activity [37] and to promote the adhesion and osteogenic differentiation of mesenchymal stem cells [38,39], so Tb^3+^:cit-cAp nanoparticles are also a promising biomimetic, lanthanide-doped apatitic filler for bone reconstruction.

The aims of the present contribution are: (i) to investigate the possibility to obtain luminescent biomimetic and highly biocompatible Tb^3+^:cit-cAp nanocrystals via the thermal decomplexing precipitation method; (ii) to study their physico-chemical, luminescence and cytocompatibility properties; and (iii) to assess nanoparticle cell uptake and the feasibility for cytoplasmatic fluorescence imaging using flow cytometry and confocal fluorescence microscopy.

In a first step, precipitates were generated by thermal decomplexing of Tb^3+^/Ca^2+^/citrate/phosphate/carbonate solutions and thoroughly characterized using a large set of complementary techniques. Special attention was paid to conditions that allowed to obtain nanoparticles with biomimetic features. Afterward, the luminescent properties of the produced particles were explored. Finally, the cytocompatibility of such particles and the possibility to use them to image cells was investigated via in vitro cell tests.

## 2. Materials and Methods

### 2.1. Reagents and Precipitation Technique

Sodium citrate tribasic dihydrate (Na_3_(cit)·2H_2_O, cit = citrate = C_6_H_5_O_7_, ACS reagent, ≥99,0% pure), terbium (III) chloride anhydrous (TbCl_3_, 99.9% pure, trace metals), calcium chloride dihydrate (CaCl_2_·2H_2_O, Bioxtra, ≥99.0% pure), and sodium phosphate dibasic (Na_2_HPO_4_, ACS reagent, ≥99.0% pure) were provided by Sigma-Aldrich (St. Louis, MO, USA). Sodium carbonate monohydrate (Na_2_CO_3_·H_2_O, ACS reagent, 99.5% pure) was supplied by Merck (Darmstadt, Germany) and hydrochloric acid (HCl, ACS reagent, 37 wt% in H_2_O) by Panreac (Barcelona, Spain). Solutions were prepared with ultrapure deionized Milli-Q water (0.22 μS, 25 °C, Millipore, Burlington, MA, USA). The precipitation experiments were carried out by thermal decomplexing of M^(2+/3+)^/Ca^2+^/cit/phosphate/carbonate solutions at 80 °C [30,31], using Tb^3+^ as doping lanthanide ion (*x* = 0, 0.001, 0.005, 0.010, 0.015, and 0.020 M) and decreasing concentrations of Ca^2+^ in the precipitating solution (*y* = 0.100, 0.099, 0.095, 0.090, 0.085, and 0.080) to set *x* + *y* = 0.100 M. The experiments lasted 4 h, 24 h, 96 h, and 7 days. The precipitates were subjected to washing by centrifugation with ultrapure water (6 cycles, 9000 rpm, 9 min each) and freeze-dried overnight at −50 °C under vacuum (3 mbar).

### 2.2. Physico-Chemical Characterizations

Precipitates were characterized using different techniques. X-ray powder diffraction (XRD) data were collected using a BrukerD8 Advance Vario diffractometer (Bruker GmbH, Karlsruhe, Germany) with a Bragg Brentano parafocusing geometry and Cu Kα1 radiation (1.5406 Å). Transmission electron microscopy (TEM) observations were performed with a TEM Libra 120 Plus (Carl Zeiss, Jena, Germany) instrument operated at 80 kV. Samples were dispersed in absolute ethanol (≥99.8% *v*/*v*) and deposited on copper microgrids coated with FORMVAR carbon film. High resolution TEM (HRTEM) and selected area electron diffraction (SAED) analyses were performed with a TITAN G2 60–300 FEI Instrument operating at 300 kV (FEI, Hillsboro, OR, USA). The instrument was equipped with an EDX Super X detector to perform elemental microanalysis and element mappings, as well as scanning transmission electron microscope (STEM) type high-angle annular dark-field imaging (HAADF).

Fourier transform infrared spectra (FTIR) were recorded in transmittance mode within a wavenumber range from 4000 cm^−1^ to 400 cm^−1^ using a Perkin-Elmer Spectrum One FTIR spectrometer (Perkin Elmer, Shelton, WA, USA). Pellets with ~1 wt% sample in anhydrous KBr were prepared and pressed using a hydraulic pump at 10 tons. Pure KBr pellets were used to record the background. Raman spectra were recorded with a LabRAMHR spectrometer (Jobin–Yvon, Horiba, Japan). The excitation line was provided by a diode laser emitting at a wavelength of 532 nm while a Peltier cooled charge-coupled device (CCD) (1064 × 256 pixels) was used as the detector.

Particle size distribution (PSD) and electrophoretic mobility (ζ-potential) were analyzed with a Zetasizer Nano ZS analyzer (Malvern Instruments Ltd., Malvern, UK) in aqueous suspensions (~0.5 mg/mL, 25 °C) in disposable polystyrene cuvettes. For ζ-potential versus pH measurements, an MPT-2 autotitrator (Malvern Instruments Ltd.) was employed to adjust the pH of the suspensions. Diluted HCl and NaOH solutions (0.25 and 0.1 M, respectively) were used as titration agents without any additional electrolytes. To analyze the effect of citrate in terms of ζ-potential versus pH measurements, we compared the values obtained for samples prepared with *x* = 0, 0.001, 0.005, and 0.010 M Tb^3+^, with those measured for the same particles prepared after immersion in a stirred 0.1 M NaOH solution for 1 h, since this procedure allowed us to remove the citrate ions [24,27].

Elemental composition of the samples was measured by inductively-coupled plasma optical emission spectrometry (ICP-OES) using an Agilent 5100 instrument (Agilent Technologies, Santa Clara, CA, USA). Before analysis, 10 mg of sample was dissolved in triplicate in 50 mL of a 1 wt% HNO_3_ aqueous solution.

The thermal behavior of the samples was studied using a simultaneous thermogravimetric differential thermal analyzer (TG-DTA), using a STA 449F3 Jupiter device (Netzsch GmbH, Selb, Germany). The samples were placed in an alumina pan, and an empty crucible was used as a reference. The heating profile was set from room temperature to 1100 °C with a ramp of 10 °C min^−1^ under airflow.

### 2.3. Luminescence Spectroscopy

The luminescence properties (excitation and emission spectra and lifetime) of solid Tb^3+^-doped particles and their aqueous suspensions (~0.5 mg/mL) were recorded using a Cary Eclipse Varian Fluorescence Spectrophotometer (Varian Australia, Mulgrave, Australia). A front surface accessory was used to obtain the luminescence spectra, the luminescence lifetime (*τ*), and the relative luminescence intensities (R.L.I.) of the powders and a commercial Peltier cell holder connected to a temperature control module (Agilent Technologies, Madrid, Spain) was used to obtain the luminescence spectra and the R.L.I. of the liquid suspensions. The instrumental parameters for measuring the excitation and emission spectra of both the powder and aqueous suspension (~0.5 mg/mL) samples were *λ_exc_* = 375 nm, *λ_em_* = 545 nm, delay time (*t_d_*) = 120 µs, and gate time (*t_g_*) = 5 ms. For powder samples, the photomultiplier voltage was of 470 V and slit width_exc/em_ 5/5 nm, and for aqueous suspension particles photomultiplier voltage was of 800 V and slit width_exc/em_ 10/10 nm. The excitation and emission spectra were recorded within wavelength ranges 250–500 nm and 500–750 nm, respectively. Luminescence lifetime measurements were also recorded using a Cary Eclipse Varian Fluorescence Spectrophotometer (Varian Australia, Mulgrave, Australia) using *λ_exc/em_* = 375/475 nm, 100 µs delay time (*t_d_*), 0.010 ms gate time (*t_g_*), photomultiplier voltage of 600 V, slit width_exc/em_ 10/10 nm, and 100 cycles.

### 2.4. Biological Tests

#### 2.4.1. Cells Culture

HeLa, human cervical cancer cell line, was obtained from ATCC. MCF7 human breast cancer and A375 melanoma cancer cell lines were obtained from Biobanco (Sistema Sanitario Público Andaluz, Granada, Spain). Human fibroblasts were isolated from skin tissue from abdominoplasty surgery using an enzymatic digestion with collagenase I (Sigma-Aldrich, St. Louis, MI, USA). Cells were grown in Dulbecco’s modified Eagle’s medium (DMEM) (Sigma-Aldrich) supplemented with 10% fetal bovine serum (FBS) (Sigma-Aldrich), and 1% penicillin/streptomycin (P/S) (Sigma-Aldrich), in an atmosphere containing 5% CO_2_ at 37 °C.

#### 2.4.2. Cell Proliferation Assays

HeLa, MCF7, A375, and fibroblast cells lines were seeded onto 96 wells plate at a confluence of 4 × 103 cells/wells in DMEM (Sigma-Aldrich) supplemented with 10% fetal bovine serum (FBS) (Sigma-Aldrich), and 1% penicillin/streptomycin (P/S) (Sigma, St. Louis, MO, USA). Plates were incubated at 37 °C in 5% CO_2_. Twenty-four hours after seeding, cells were treated with 100, 10, 1, and 0.1 µg/mL Tb-containing samples prepared with *x* = 0.001, 0.010, 0.015, and 0.020 M Tb^3+^. Additionally, a non-doped sample was added (undoped). Seventy-two hours after treatment, the medium was removed and 100 µL of MTT (3-(4,5-dimethylthiazol-2-yl)-2,5-diphenyltetrazolium bromide) was added. After 3 h of incubation at 37 °C and 5% CO_2_, the MTT was removed and 100 µL DMSO were added. Immediately, the plate was measured using an absorbance spectrophotometer at 570 nm.

#### 2.4.3. Confocal Microscopy

Cell line A375 was seeded in an eight-well chamber slide (Ibidi, Gräfelfing, Germany) at a density of 8000 cells/well. They were treated after 24 h with Tb^3+^-containing samples (*x* = 0.01 M and 0.02 M Tb^3+^) at 100 µg/mL for 12 h and 24 h. Next, cells were fixed in paraformaldehyde (PFA) at 4% for 20 min at room temperature and washed with PBS. Images were obtained using a spectral confocal Leica TCS SP2 AOBS microscope.

#### 2.4.4. Flow Cytometry

Cell lines were treated with Tb^3+^-doped samples (*x* = 0.01 M and 0.02 M Tb^3+^) at 100, 10, 1, and 0.1 µg/mL during 12 h and 24 h. Afterwards, cells were fixed with PFA 4% for 20 min and washed with PBS. Cells were then processed with a FACScan flow cytometer (Becton Dickinson, San Jose, CA, USA).

## 3. Results

### 3.1. Crystallographic, Compositional, Morphological and Spectroscopic Features of the Precipitates 

**Figure 1:**
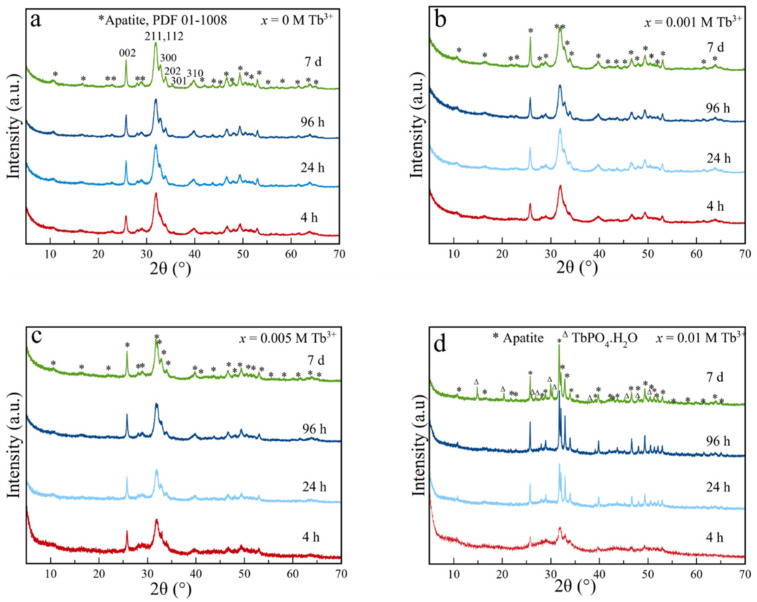
X-ray diffraction patterns of precipitates at 4 h, 24 h, 96 h, and 7 days, and at different x: (**a**) 0, (**b**) 0.001, (**c**) 0.005, and (**d**) 0.010 M Tb^3+^. * (apatite, PDF 01-1008); ^Δ^ (TbPO4·H_2_O, PDF 20-1244).

The XRD patterns of Tb^3+^-free (*x* = 0) and Tb^3+^-doped samples prepared with nominal Tb^3+^ doping concentrations from *x* = 0.001 to 0.010 M are plotted in Figure 1. All samples up to *x* = 0.005 M Tb^3+^ show a similar evolution with time. At 4 hours of maturation, the major peaks of hydroxyapatite phase are already present (PDF 01-1008), i.e., at 2*θ* 25.87° (plane (002)), 31.77° (211), 32.19° (112), 32.90° (300), 33.9° (202), and 39.81° (310) [23,30]. The high broadness of the peaks indicates that the particles are nanocrystalline.

For *x* = 0.010 M Tb^3+^, the evolution is different. At 24 h, the diffractogram presents narrower peaks characteristic of a more crystalline material with larger dimensions and at 7 days, in addition, are also present the peaks of hexagonal TbPO_4_·H_2_O (rhabdophane, space group P3121, PDF 20-1244). By increasing *x* to 0.015 and 0.020 M Tb^3+^ (see Supplementary Materials, Figure S1), the baseline of the XRD patterns at all maturation times shows bulging, suggesting the presence of an amorphous phase. This profile is similar to the pattern of amorphous TbPO_4_·*n*H_2_O, suggesting that this phase is dominant at lower maturation times [40]. At 7 days, the rhabdophane phase, instead, was formed for both 0.015 and 0.020 M Tb^3+^ concentrations.

The chemical composition of most matured precipitates (Table 1) revealed that the progressive increase of x induced an increment of Tb content and a decrease of Ca and P, while there were almost no differences in terms of function of time. The (Ca + Tb)/P molar ratio (≈1.60–1.50) of samples composed only of apatite (*x* < 0.010 M, 96 h) was lower than that of stoichiometric hydroxyapatite (Ca/P = 1.67). The calcium deficiency of the samples is expected, as is characteristic of biomimetic apatites. For samples prepared with higher *x*, the (Ca + Tb)/P ratios were lower due to the presence of TbPO_4_ phases.

The thermogravimetric curves are shown in Figure S2a,b of the Supplementary Materials. Except for *x* = 0.015 M and *x* = 0.020 M at 96 h, all samples displayed a TGA profile similar to that reported for other biomimetic carbonate apatites prepared using the same method [27]. The curves present four main weight losses, marked as peaks in the first derivative of the thermogram (DTG, Figure S2c), which correspond to (i) the loss of adsorbed water (from room temperature to ≈ 220 °C), (ii) the loss of tightly bound, structural water (from ≈ 220 °C to ≈ 380 °C), (iii) the loss due to the decomposition of adsorbed citrate molecules (from ≈ 380 °C to ≈ 600 °C), and (iv) the loss due to the decomposition of carbonates in CO_2_ (from ≈ 600 °C to ≈ 1100 °C). In samples *x* = 0.015 M and *x* = 0.020 M Tb^3+^, especially at 96 h, the curves present additional weight losses due to the presence of the amorphous TbPO_4_·*n*H_2_O phase. For these samples, the contents of water, citrate, and carbonate could not be accurately assessed; thus, for the sake of comparison we have given data only for samples prepared at 7 days. Table 1 shows the content of water, citrate, and carbonate of most matured samples. It was observed that the water content (both adsorbed as humidity and structural) increased with *x*, particularly in samples *x* > 0.010 M Tb^3+^, due to the presence of hydrated TbPO_4_ phases. However, the citrate and carbonate content showed little variability. This could be due to the presence in these precipitates of hydrated TbPO_4_ in both amorphous and rhabdophane phases. With maturation, for the samples *x* = 0.001 M and *x* = 0.005 M Tb^3+^ there is no variation in water, citrate, or carbonate content between 96 h and 7 days, suggesting that progressive apatite crystallization did not affect the chemical composition. On the other hand, in sample *x* = 0.010 M Tb^3+^ at 7 days there was a slight increase of Ca, P, and Tb content paired with a decrease of adsorbed water content in comparison to 96 h sample (Figure S2a,b). It is likely that, with maturation, the formation of rhabdophane phase from amorphous TbPO_4_·*n*H_2_O has involved a dehydration. Regarding samples *x* = 0.015 M and *x* = 0.020 M Tb^3+^, even if the weight losses cannot be attributed with certainty, it can be observed that an intense, multistep weight loss from 500 °C to 600 °C is not present at 7 days of maturation, suggesting that it is correlated to the evolution from amorphous TbPO_4_·*n*H_2_O to rhabdophane phase.

**Figure 2:**
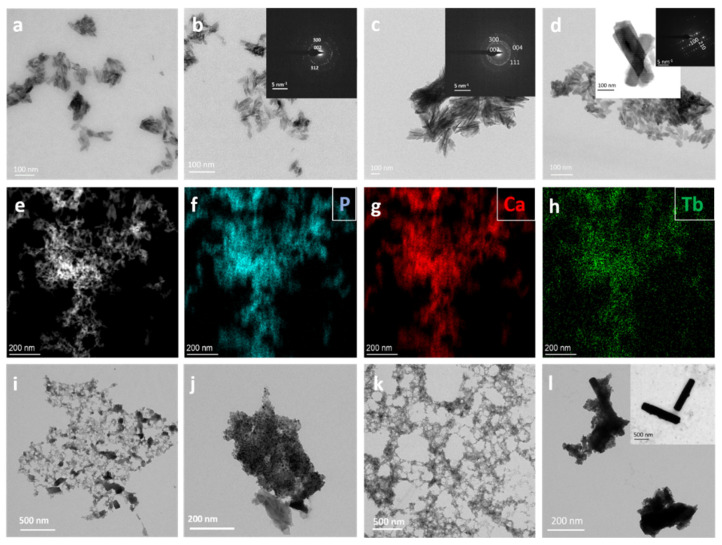
TEM images of Tb^3+^:cit-cAp particles prepared at different nominal Tb^3+^-doping concentrations (*x*) and maturation times: (**a**) *x* = 0 M, 96 h; (**b**) *x* = 0.001 M, 96 h; (**c**) *x* = 0.005 M, 96 h, insets show SAED patterns; (**d**) *x* = 0.010 M, 4 h; inset *x* = 0.010 M, 96 h, shows apatite prisms and the SAED pattern; (**e**) *x* = 0.001 M, 96 h, HAADF image; (**f**–**h**) EDX element mapping analysis of P, Ca and Tb; (**i**) *x* = 0.015 M, 4 h, (amorphous particles); (**j**) *x* = 0.015 M, 96 h, TbPO_4_·H_2_O, amorphous TbPO_4_·*n*H_2_O, and apatite; (**k**) *x* = 0.020 M, 4 h, amorphous particles; (**l**) *x* = 0.020 M, 96 h, apatite prisms and TbPO_4_·H_2_O nanoparticles; inset shows apatite prisms at 7 days.

Figure 2 shows TEM micrographs of Tb^3+^:cit-cAp nanoparticles. For the samples prepared with *x* ≤ 0.005 M at 96 h of maturation (Figure 2a–c), the particles presented needle-like crystal habits with length of 40–80 nm. Elemental EDX mapping of the lowest doped nanocrystals (*x* = 0.001 M Tb^3+^, 96 h) shows a homogeneous composition of P, Ca and Tb (Figure 2e–h). The micrographs of Tb^3+^:cit-cAp prepared with *x* = 0.010 M Tb^3+^ show 50–70 nm dispersed nanoapatites at 4 h (Figure 2d), and prismatic crystals of apatite up to ≈300 nm at 96 h (Figure 2d, inset). For *x* = 0.005 M and 0.010 M Tb^3+^ at 96 h the particles started to aggregate (Figure 2c and inset of Figure 2d), and at 7 days of maturation the prisms formed spherical agglomerates (see Figure S3a,b,d,e in the Supplementary Materials), which, in the case of *x* = 0.010 M Tb^3+^, coexist with rhabdophane nanoparticles.

When *x* is increased to 0.015 M Tb^3+^, the sample is amorphous at 4 h (Figure 2i), while at 96 h (Figure 2j) it contains apatite prisms, as well as amorphous TbPO_4_·*n*H_2_O nanoparticles. At 7 days, aggregates of apatite prisms coexist with TbPO_4_ nanoparticles, both as amorphous and as rhabdophane phases (Figure S3c,f). The same was found at *x* = 0.020 M Tb^3+^ (Figure 2k,l). At this *x* value, the apatite prims had lengths up to ~1.5 µm. The combined results of XRD and TEM indicate that optimal maturation times to obtain nanosized apatites are less than 7 days for *x* = 0.001 M Tb^3+^, less than 96 h for *x* = 0.005 M Tb^3+^, and 4 h for *x* = 0.010 M Tb^3+^.

**Figure 3:**
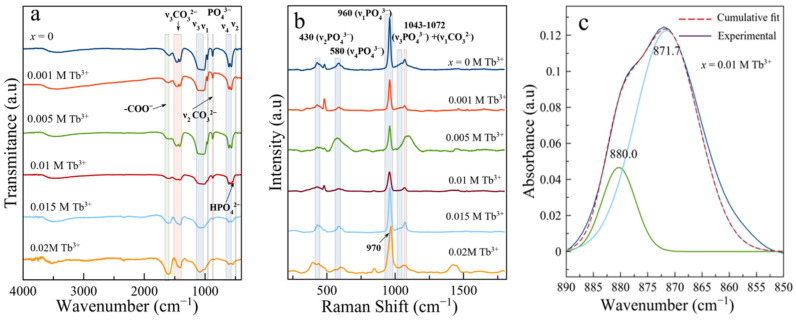
(**a**) FTIR and (**b**) Raman spectra of samples at 96 h maturation varying *x* (from 0 to 0.02 M Tb^3+^), (**c**) deconvolution of the υ_2_CO_3_ region of the sample *x* = 0.01 M Tb^3+^ 96 h.

Figure 3, Figures S4 and S5 of the Supplementary Materials show FTIR and Raman spectra of the samples. All FTIR spectra of Tb-free (*x* = 0) and Tb-doped apatite specimens in the *x* range from 0.001 to 0.010 M Tb^3+^, up to 96 h, exhibited broad O–H stretching of physisorbed water between 3600 cm^−1^ and 2800 cm^−1^. The apatitic OH bands around 3580 cm^−1^ and 620 cm^−1^ were not clearly detected, as is usually found in bio-inspired apatites. The 400–1800 cm^−1^ region displays the typical spectral features of apatitic compounds, i.e., the asymmetric stretching mode of PO_4_^3−^ groups (υ_3_PO_4_) at 1000–1100 cm^−1^ (Figure 3a and Figure S4), the symmetric stretching υ_1_PO_4_ at υ958–960 cm^−1^ (small band), the bending modes υ_4_PO_4_ at ~608, and 564 cm^−1^ and υ_2_PO_4_ at ~470 cm^−1^. The shoulder at ~535 cm^−1^ in the υ_4_PO_4_ domain is attributed to surface HPO_4_^2−^ (non-apatitic) and points to the biomimetic character of these compounds [41].

The presence of carbonate (CO_3_^2−^) is attested by the bands at ~1414 cm^−1^ and 1473 cm^−1^ (υ_3_CO_3_), and the bulging peak around 873 cm^−1^ (υ_2_CO_3_). From the area ratio between the υ_3_CO_3_ and the υ_1_υ_3_PO_4_ contributions (r_c/p_) the estimated overall degree of carbonation [42] for sample *x* = 0.010 M at 96 h is 4.02 ± 0.5 wt%, which fully agrees with the value determined by TGA. This degree of carbonation is slightly lower than that determined (up to 5.9 wt%) in Tb^3+^-free samples [23]. 

In the doped samples (i.e., *x* = 0.010 M Tb^3+^) the deconvoluted υ_2_CO_3_ region (Figure 3c) display the peaks at ∼880 and ∼872 cm^−1^, which correspond to A- and B-type substitutions, where carbonate replaces OH^−^ and PO_4_^3^^−^, respectively [14]. In addition to these signals, all samples displayed a band at ∼1590–1600 cm^−1^, ascribed to the antisymmetric stretching frequency of -COO^−^ groups of the citrate [43].

Complementary spectroscopic characterization by Raman (Figure 3b and Figure S5 of the Supplementary Materials) shows the characteristic apatitic peak at ~960 cm^−1^ (υ_1_PO_4_) [44], as well as the peaks at ~430 cm^−1^ (υ_2_PO_4_) and ~530 cm^−1^ (υ_4_PO_4_). The signal at ~1072 cm^−1^ is due to a combination of the υ_1_CO_3_ mode at 1070 cm^–1^ with the υ_3_PO_4_ mode at 1076 cm^–1^ [45]. The small peak at 845 cm^−1^, clearly visible in Figure S5c–e of the Supplementary Materials, is assigned to the δCOO^−^ mode of citrate ions [23]. For *x* = 0.015 M at 4 h of maturation (Figure S5e) and *x* = 0.020 M at every maturation time (Figure S5f), the υ_1_PO_4_ signal is shifted at ~965–970 cm^−1^ likely due to the contributions of amorphous TbPO_4_·*n*H_2_O and rhabdophane in the sample [33,44].

### 3.2. Particle Size Distribution and ζ-Potential of Nanocolloids

The PSD and ζ-potential of colloidal particles at pH of physiological interest are useful parameters to evaluate the suitability of the particles as nanocarriers in nanomedicine. Indeed, the dispersion/aggregation behavior of the colloid at pH around 7.4 (representing the blood) or pH 5–6 (representing the tumor microenvironment) depends on the size and surface charge of the particles [14,30]. These characteristics also affect the creation of the protein corona around the particles within the biological plasma [46].

**Figure 4:**
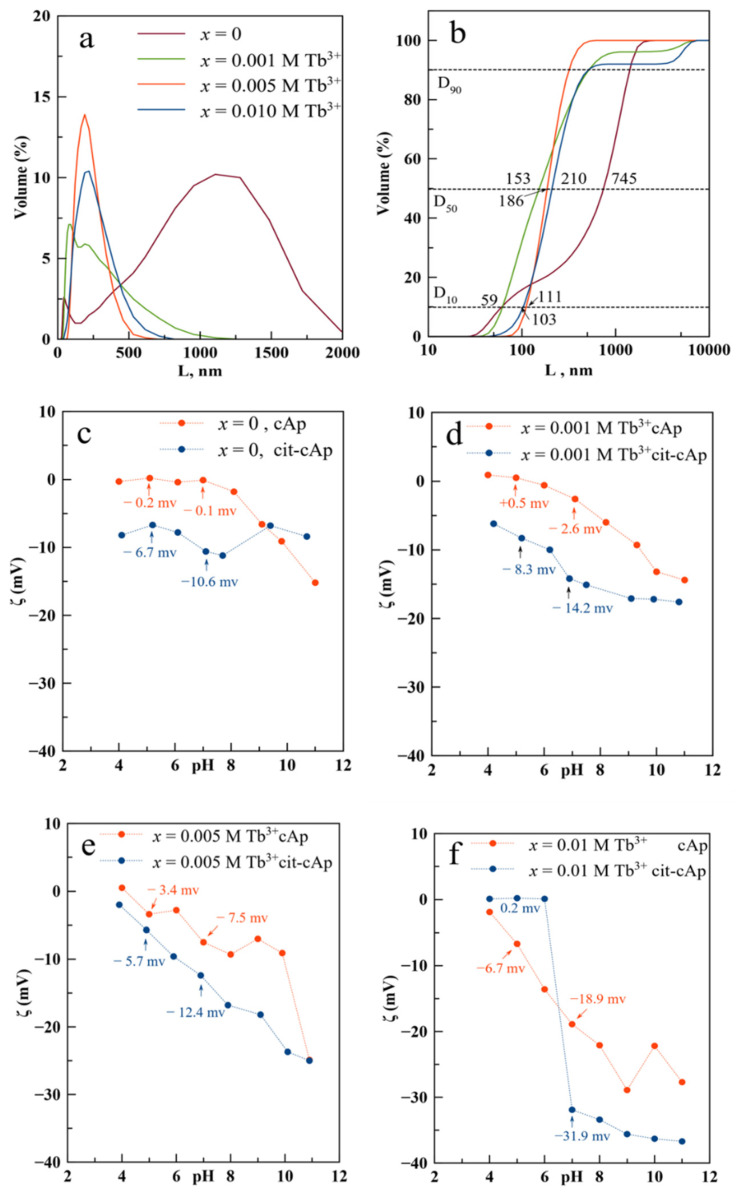
(**a**) Volume based PSD, (**b**) cumulative volume based PSD, and (**c**–**f**) ζ-potential versus pH of Tb^3+^:cit-cAp and Tb^3+^:cAp nanoparticles prepared with Tb^3+^ doping concentrations *x* = 0, 0.001, 0.005 and 0.010 M for 96 h.

In this study the PSD of Tb^3+^:cit-cAp was plotted both as volume distribution (Figure 4a) and as cumulative volume-based distribution (Figure 4b), to reveal the percentiles D_10_, D_50_ and D_90_ of the population. These values illustrate the percentage of cumulative volume undersize distribution (percentage of the crystals smaller than the indicated size). Accordingly, D_10_ is closer to the individual particle size whereas D_50_, the median size, is to some extent influenced by particle aggregation and D_90_ is completely dominated by aggregates. The percentiles corresponding to samples *x* = 0, 0.001, 0.005, and 0.010 M Tb^3+^ at 96 h were D_10_ 59, 59, 111, and 103 nm, respectively, and D_50_ 745, 186, 153, and 210 nm, respectively (Figure 4b). The PSD for Tb^3+^-free nanoparticles is more influenced by aggregation compared to Tb^3+^-doped nanoparticles (Figure 4b).

To assess the effect of surface citrate and Tb^3+^ doping on the aggregation/dispersion behavior, we have compared the curves of ζ-potential versus pH (Figure 4c–f) of Tb^3+^:cit-cAp with those of the same particles after removing citrate (Tb^3+^:cAp). An inversed correlation between ζ-potential and pH was observed. In addition, Tb^3+^:cit-cAp samples always had more negative ζ-potential values in comparison to Tb^3+^:cAp. At higher *x* values, the inverse correlation between ζ-potential and pH was stronger. For example, at pH 7 the increase of *x* from 0 to 0.010 M Tb^3+^ induced a ζ-potential decrease from 0 to −18.9 mV for Tb^3+^:cAp samples and from −10.6 to −31.9 mV in Tb^3+^:cit-cAp particles. These strongly negative ζ-potential values favor the dispersion of both types of colloids. However, the increase in *x* had little impact on the ζ-potential at pHs of 5–6. We can conclude that citrate and Tb^3+^ doping act synergistically to decrease the ζ-potential, particularly at pH ≥ 7.

### 3.3. Luminescence Properties in Solid State and in Aqueous Suspensions

**Figure 5:**
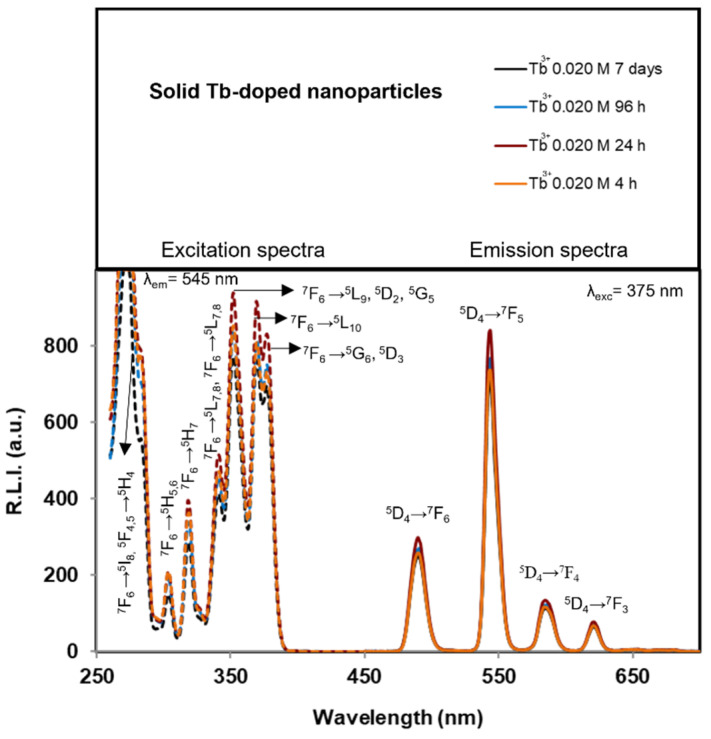
Uncorrected excitation (dashed lines) and emission (solid lines) spectra of solid samples prepared with *x* = 0.020 M Tb^3+^ at maturation times of 4 h, 24 h, 96 h and 7 days.

**Figure 6:**
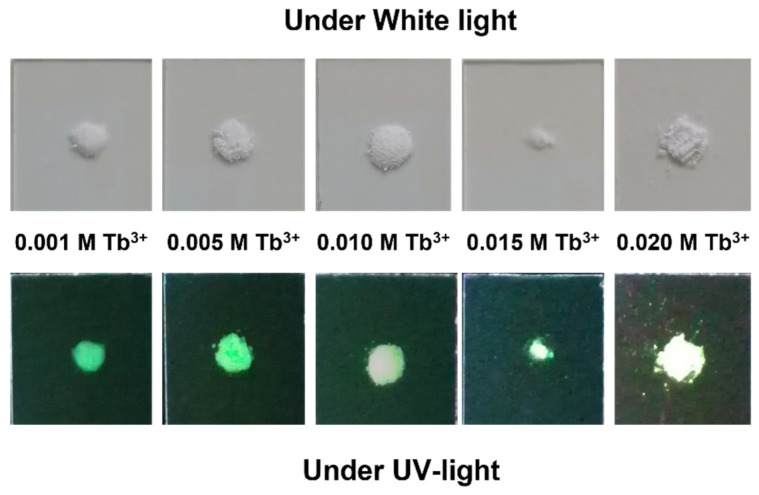
Pictures of samples prepared with different Tb^3+^ doping concentrations (*x*) at maturation times of 96 h under sunlight (**top**) and 254 nm UV-lamp illumination (**bottom**).

The luminescence properties of the particles are shown in the Supplementary Materials (see Figures S6 and S7). The *λ_exc_* and *λ_em_* are the same as those depicted in Figure 5. The observed *λ_exc_* were 230, 283, 302, 319, 340, 352, 369, and 375 nm. The broad bands between 200 and 300 nm, centered at 230 nm, correspond to charge transfer bands (CTB), which occur by electron delocalization from the filled 2p shell of O^2–^ to the partially filled 4f shell of Tb^3+^. In addition, this band can be partially attributed to the charge transfer transition X^5+^–O^2^^–^ [47,48]. The remaining less intensive excitation wavelengths correspond to the ^7^F_6_→^5^I_8_ and ^5^F_4,5_→^5^H_4_, ^7^F_6_→^5^H_5,6_, ^7^F_6_→^5^H_7_, and ^7^F_6_→^5^L_7,8_, ^7^F_6_→^5^L_9_,^5^D_2_,^5^G_5_, ^7^F_6_→^5^L_10_, and ^7^F_6_→^5^G_6_,^5^D_3_ transitions [49]. Figure 6 shows the naked-eye aspect of the solid materials (96 h) under white and UV lights, where the green emission of the Tb^3+^ is visible

Figure S6 (see Supplementary Materials) shows the emission spectra of particles prepared in solutions with *x* = 0.020 M Tb^3+^ at 96 h using *λ_ex_* = 230 (UV) and 375 nm (near visible). The emission spectra are the same and only the relative intensities being affected by *λ_ex_*. Thus, for nanomedicine applications, we selected *λ_exc_* = 375 nm, as it is close to the visible spectra. 

Concerning the *λ_em_*, the emission bands are centered at 491, 545, 585 and 621 nm, which correspond to the Tb^3+ 5^D_4_→^7^F_6_, ^5^D_4_→^7^F_5_, ^5^D_4_→^7^F_4_ and ^5^D_4_→^7^F_3_ transitions, respectively [50]. As the *λ_em_* corresponding to the hypersensitive transition without an inversion center (^5^D_4_→^7^F_5_, 545 nm for Tb^3+^) produces the highest R.L.I., we selected this *λ_em_* for the study.

Figures S8 and S9 (see Supplementary Materials) exhibit the effect of maturation time at different *x* on the R.L.I. Only samples *x* = 0.010 M Tb^3+^ show a significant variation of the R.L.I. with maturation time while the rest of samples with *x* < 0.010 M Tb^3+^ show a time-independent R.L.I. This difference can be due to the existence of rhabdophane, which shows lower luminescence emission [33]. This phase is also present on samples *x* = 0.015 M and *x* = 0.020 M Tb^3+^ of 7 days of maturation but in lower amounts; in addition, these materials contain the amorphous TbPO_4_·*n*H_2_O phase which contributes positively to the R.L.I. [33].

Analyzing the evolution of the R.L.I at a fixed maturation time versus *x* it is observed that the increase of *x* increases the luminescence in all cases with a linear trend, as expected [30].

**Figure 7:**
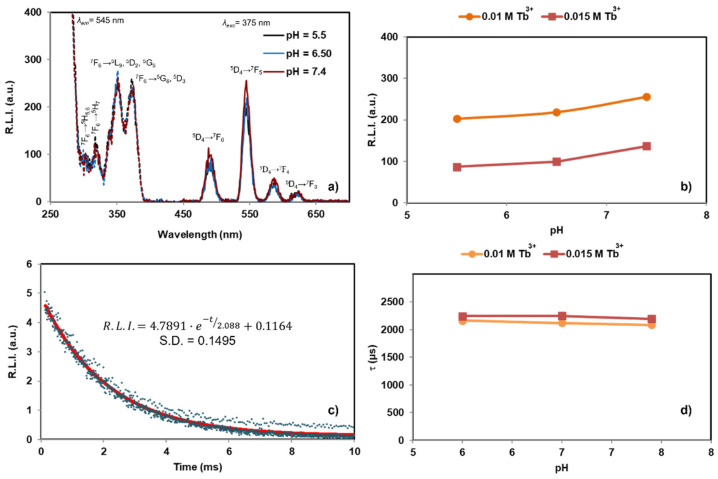
Luminescence properties of samples (*x* = 0.015 M Tb^3+^, *t* = 96 h) dispersed in aqueous media at 25 °C: (**a**) excitation (dashed line) and emission (solid line) spectra for samples *x* = 0.015 M Tb^3+^ at different pHs; (**b**) evolution of R.L.I. with pH; (**c**) luminescence decay curve of sample *x* = 0.015 M Tb^3+^ at pH = 7.4; and (**d**) evolution of *τ* versus pH.

Concerning the luminescence lifetime (*τ*), in Supplementary Materials (see Figures S10–S12) are reported the luminescence decay curves and the variation of *τ* versus both maturation time and *x*, respectively. It is shown that, only when *x* = 0.010 M Tb^3+^ does the maturation time affect *τ* (see Supplementary Materials, Figure S11). This variation can be caused by the presence of rhabdophane in the precipitate at 7 days. On the other hand, *τ* for a given maturation time slightly decreased when *x* increases from 0.005 M to 0.010 M Tb^3+^, and might be due to the increase of size and crystallinity of the Tb^3+^:cit-cAp samples at higher *x*. For the highest Tb^3+^ concentrations (*x* = 0.015 and 0.020 M Tb^3+^), *τ* is practically the same. The luminescence properties of all samples in aqueous suspensions are similar to those depicted in Figure 7. The luminescence properties (*λ_exc_*, *λ_em_*, R.L.I. and *τ*) are the same at any of the tested pHs, so it is likely that changing the pH in a biological environment does not affect these properties.

The effect of the ionic strength on the luminescence properties (see Supplementary Materials, Figure S13) shows that neither the R.L.I. nor *τ* were significantly affected, which is rather important for nanomedical applications.

Figure S14 of the Supplementary Materials shows the effect of the temperature. In brief, increasing from 25 to 40 °C does not affect significantly the R.L.I. of the suspended particles. The variation in the luminescence intensity is normally 1% per °C [51], however for these materials the decreases were 2.1 and 0.7% per °C for *x* = 0.01 and 0.015 M Tb^3+^ respectively. This is particularly important in view of in vivo imaging (~37.4 °C) as the rest of experiments were performed at room temperature (25 °C).

### 3.4. Biological Tests

#### 3.4.1. Cytocompatibility of the Particles

**Figure 8:**
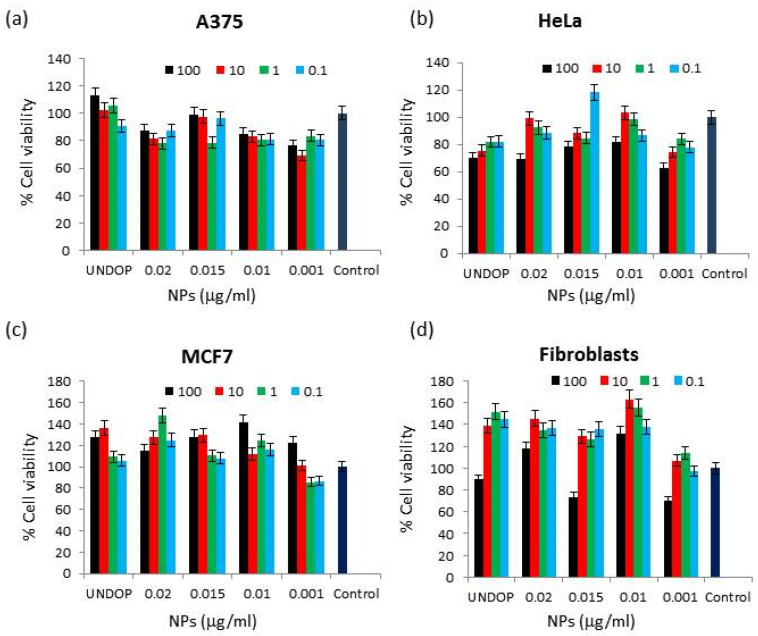
MTT assays results carry out with Tb^3+^containing nanoparticles on A375, HeLa, MCF7 and Fibroblasts cells. A375 (**a**), Hela (**b**), MCF (**c**), Fibroblasts (**d**) cells. Data is displayed as control cells compared to cells treated with nanoparticles prepared at *x* = 0.020, 0.010, 0.015, and 0.001 M Tb^3+^. The data are presented as means ± s.d. (*n* = 3) from three independent experiments using two-way ANOVA.

The biological effects of Tb^3+^:cit-cAp nanoparticles at different concentrations were tested in three carcinogenic cells lines (A375, MCF7, and HeLa) and in a non-carcinogenic cell line (Fibroblast). As expected, the nanoparticles did not show cytotoxic effects; only a decrease in confluence appeared at the highest concentrations, but cell viability was always greater than 70% (Figure 8).

As shown in Figure 8, all samples were cytocompatible. In addition, cell viability was not affected by the increase in Tb^3+^ doping, as was corroborated by statistical analysis where no significant differences in cell growth were obtained, nor in the treated, nor in control tumor lines. In the healthy cell line, although a more marked decrease in cell viability was observed at a concentration of 100 µg/mL, cell viability always remained higher than 70% without statistically significant differences compared to the control (*p*-value doping Tb^3+^ = 0.907, concentration *p*-value = 0.767).

**Figure 9:**
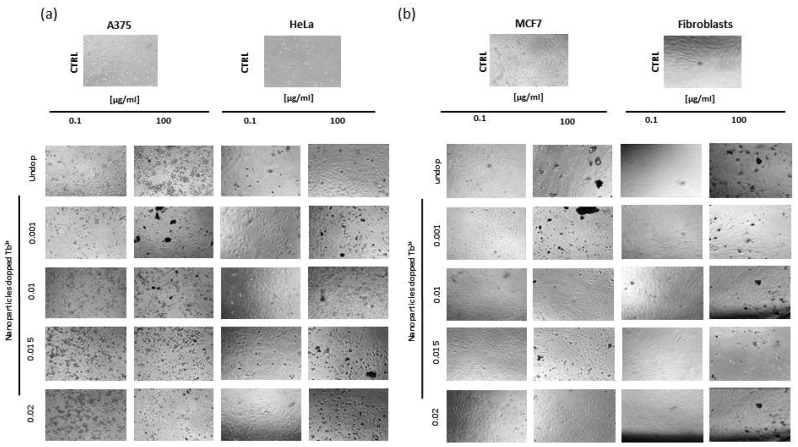
Representative microscopy images of control cells against treated cells with particles prepared with *x* = 0, 0.001, 0.01, 0.015 and 0.02 M Tb^3+^ in (**a**) A375 and HeLa, and (**b**) MCF7 and Fibroblasts cell lines. All images are at 10× magnification.

Figure 9 shows that there were no morphological changes between untreated cells and those treated with Tb^3+^:cit-cAp nanoparticles with different Tb^3+^ doping, confirming the MTT results. Our results are similar to those previously obtained by using Eu^3+^:cit-cAp [30].

#### 3.4.2. Flow Cytometry: Uptake and Intracellular Localization

**Figure 10:**
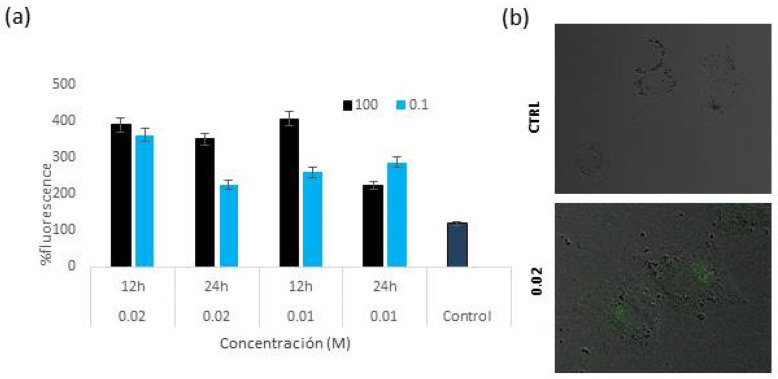
Uptake and intracellular localization of Tb^3+^:cit-cAp nanoparticles. (**a**) Uptake of particles prepared with *x* = 0.010 M and 0.020 M Tb^3+^ at 100 µg/mL and 0.1 µg/mL at 12 h and 24 h after treatment. (**b**) Merged confocal fluorescence and phase contrast microscopy images (20×) of A375 cells incubated for 12 h with Tb^3+^-doped particles. Intracellular cytoplasmic uptake can be observed at 12 h after treatment with Tb^3+^ containing particles (*x* = 0.020 M Tb^3+^).

Flow cytometry was used in order to verify the luminescence characteristics, uptake and intracellular localization of the particles. The experiment was carried out using A375 cell line that showed 80% viability for the treatment with particles prepared with *x* 0.010 M and 0.020 M Tb^3+^ at 96 h. We decided to analyze concentrations at 100 µg/mL and 0.1 µg/mL at two different times: 12 h and 24 h (Figure 10).

The intensity of cell fluorescence increased in all cases compared to the untreated control, the highest being for the particles prepared with *x* = 0.010 M Tb^3+^ at a concentration of 100 µg/mL at 12 h of treatment. At 24 h, the fluorescence decreased (Figure 10a). Moreover, cell internalization was further demonstrated by confocal microscopy as we can see in merged confocal fluorescence and phase contrast microscopy images observation (Figure 10b).

## 4. Discussion

The above findings confirm that the herein reported bioinspired route succeeded in preparing Tb^3+^: cit-cAp nanocrystals of 40–80 nm in length when Tb^3+^ doping concentration in the mother solutions was *x* ≤ 0.005 M with optimal maturation times below 7 days. Nanocrystals with Tb^3+^ content as high as 1.59 ± 0.04 wt% (for *x* = 0.001 M, 96 h) and 7.33 ± 0.13 wt% (for *x* = 0.005 M, 96 h) were obtained in these conditions. After 96 h of maturation, aggregation and agglomeration prevailed and the most mature crystals were found forming sheaf wheat or spherical agglomerates. The formation of sheaf wheat is a previous step in the formation of spherical shapes following the mechanism of spherulitic crystal growth [52]. For *x* = 0.010 M Tb^3+^, nanocrystals were produced only at 4 h of maturation. At higher times (96 h) the method yielded Tb^3+^:cit-cAp prisms up to 300 nm length, containing 12.80 ± 0.18 wt% Tb^3+^, and a carbonate content of 4.02 ± 0.18 wt%. The Tb^3+^ content of the particles is in agreement with those previously reported for Tb^3+^-doped apatites prepared using the sol-gel method, i.e., 8 and 12 wt% [34], or from 2 to 12 wt% [51], and in hydrothermal conditions at 200 °C [53]. At 7 days of maturation, the agglomeration process of Tb^3+^:cit-cAp prisms prevailed, leading to spherical agglomerates and, in addition, TbPO_4_·H_2_O nanocrystals formed, raising the total Tb^3+^ content of the precipitate to 14.20 ± 0.16 wt%. Increasing *x* to 0.015 M, the amorphous phase was found at 4 h, while the Tb-doped apatite started to appear at 24 h, and the TbPO_4_·H_2_O (rhabdophane) nanoparticles at 7 days. At *x* = 0.020 M Tb^3+^ the amorphous phase persisted at all maturation times, while Tb^3+^:cit-cAp microcrystals and TbPO_4_·H_2_O nanocrystals formed at 96 h. The higher Tb^3+^ content of these precipitates, i.e., 19.10 ± 0.40 wt% for *x* = 0.015 M Tb^3+^ and 21.71 ± 0.77 for *x* = 0.020 M Tb^3+^, is explained by the simultaneous presence of amorphous TbPO_4_·nH_2_O and rhabdophane phases besides the highly-doped Tb^3+^:cit-cAp microcrystals. 

The findings suggest that the maximum Tb^3+^ content of the apatite is ~12 wt%, and the heterogeneity of the samples (i.e., the presence of additional Tb^3+^-containing phases) is beneficial to increase the Tb^3+^ content, thus increasing the luminescence intensity. In fact, R.L.I. was almost linearly dependent on *x*, and thus, on the global Tb wt% of the precipitates, regardless the sample composition. In this respect, when *x* ≤ 0.010 M, Tb^3+^:cit-cAp nanocrystals reached an R.L.I. ~300, and for *x* > 0.010 M Tb^3+^ the R.L.I. reached 850. The presence of amorphous TbPO_4_·*n*H_2_O contributed positively to this significant increase, as previously reported [30,32]. In addition, irrespective of the nature and crystallinity of the precipitates, cell viability always remained higher than the cut-off of 70% indicated by ISO 10993–5:200970 [54], proving their high cytocompatibility. More importantly, the luminescence intensity of these particles was enough to allow intracellular localization in experiments of flow cytometry, thus demonstrating their ability as luminescent probes for bioimaging.

A key point to discuss is the nature of the amorphous phase observed in the samples with highest Tb^3+^ content. This phase was identified as amorphous TbPO_4_·*n*H_2_O, likely doped with Ca^2+^ ions by considering the following arguments: a) the bulging of the XRD baseline is similar to that reported for amorphous rare earth phosphates [40], and quite different from the one produced by amorphous calcium phosphate (ACP [27]); b) the most intense Raman signal falls at ~967 cm^−1^ (υ_1_PO_4_), similarly to found in [33], while for ACP it falls at 950–952 cm^−1^ [44]. Nevertheless, the emerging of apatite peaks from the bulging XRD baseline after 24 h of maturation for *x* = 0.015 M Tb^3+^, and at 96 h for *x* = 0.020 M Tb^3+^, might also indicate the presence in the mixture of an ACP, likely doped with Tb^3+^, acting as precursor of the apatitic phase. The presence of Tb^3+^-doped ACP and amorphous TbPO_4_·*n*H_2_O in different proportions in the amorphous mixture can explain the increase in R.L.I. when increasing *x* from 0.015 M Tb^3+^ to 0.020 M Tb^3+^ at 4 h of maturation time. The co-precipitation of terbium phosphate and apatite for *x* ≥ 0.010 M Tb^3+^ in such Ca^2+^-rich solutions is possible, because the ionic activity product largely exceeds the solubility product of both compounds. The saturation indices (S.I.) for TbPO_4_ were 10.11 for *x* = 0.010 M, 10.6 for *x* = 0.015 M Tb^3+^, and 10.40 for *x* = 0.020 M Tb^3+^. For the same solutions, the S.I.s of apatite were 17.5 for *x* = 0.010 M, 17.3 for *x* = 0.015 M Tb^3+^ and 17.2 for *x* = 0.020 M Tb^3+^ [55]. 

Concerning the impact of citrate and Tb^3+^ content on the ζ-potential, the main findings were: the important decrease of ζ-potential in function of pH in all samples as well as the more negative ζ-potential values for particles with adsorbed citrate (Tb^3+^:cit-cAp) in comparison to citrate-free ones (Tb^3+^:cAp). Finally, (the increase of Tb^3+^ doping also led a decrease in ζ-potential. The results suggest a complex role of precursor ions in this system in determining ζ-potential of the particles. According to Somasundaram [56], for salt-type minerals that are sparingly soluble and, at the same time, are reactive toward the solvent, both the solvent constituting ions and lattice ions, as well as their complexes with the solvent species are all possible ζ-potential determining ions. This means that the adsorption of these ionic species on the particle surface might influences the ζ-potential. For Tb^3+^:cAp particles the relevant species are Ca^2+^, Tb^3+^, H^+^, OH^−^, HCO_3_^−^/CO_3_^2−^, phosphates, and their charged complexes. Since the ζ-potential at pHs of 4–6 was almost zero (isoelectric point), it is possible to deduce that at these pH values Ca^2+^ and Tb^3+^ had a negligible influence in the ζ-potential, as these species are occupying crystal lattice positions and were not adsorbed to the particle surface. Only H^+^ adsorption influenced the ζ-potential at these pHs. At pHs higher than the isoelectric point, the phosphates, HCO_3_^−^/CO_3_^2−^, OH^−^ and their negatively charged complexes were likely the responsible for the decrease of the ζ-potential. For Tb^3+^:cit-cAp, it is assumed that citrate coating acted as a barrier for the dissolution of the particles. For these particles, besides OH^−^, the deprotonated –COO^−^ groups of adsorbed citrate molecules, pointing upward toward the solution, were the ones responsible for the decrease of the ζ-potential. In a previous study [57] we put forward an adsorption model describing how citrates interact with the apatite surface by ionic exchange of phosphates with citrate ions at the solid-solution interface. Depending on pH the deprotonation of the three carboxylic groups of the citric acid run according to their pKs (pK_1_ = 3.13, pK_2_ = 4.76, and pK_3_ = 6.40), allowing the citrate species to interact with the apatite in different ways. Thus, the cit^3+^ ions interact weakly in a bidentate manner (1 citrate per 2 Ca sites), whereas Hcit^2−^ interacts strongly in a monodentate manner (1 citrate per 1 Ca site). For both citrate species, the free –COO^−^ groups pointing toward the solution are those influencing the ζ-potential. The synergistic effect of Tb^3+^ doping with citrate coating is, thus beneficial for the stability of suspensions of these particles in physiological conditions, and thus for their in vivo applications.

Summing up, all the above properties are positive relative to the applications of these nanoparticles as luminescent labeling agents, or as nanocarriers for drug delivery, and highlight the potential of the thermal decomplexing synthetic method to prepare this kind of nanophosphor.

## 5. Conclusions

The citrate-based thermal decomplexing method succeeded in preparing biomimetic (bonelike) Tb^3+^:cit-cAp nanocrystals in a range of Tb^3+^ doping concentrations and maturation times, with a maximum Tb^3+^ content of ~12.8 wt% (*x* = 0.010 M Tb^3+^, 96 h). Tb^3+^ content increased to 19.10 wt% for mixtures with TbPO_4_ phases (*x* = 0.015 M Tb^3+^, 96 h) and to 21.71 wt% for mixture *x* = 0.020 M Tb^3+^, 96 h. The R.L.I. increased almost linearly with Tb^3+^ content, regardless of the sample composition, thus revealing that the presence of TbPO_4_ phases in the sample is beneficial to increase luminescence. The particles showed good luminescence properties, large Stokes shifts, high luminescence lifetime, similar optical properties in powder and dispersed in water, unaffected by pH (at physiological pH), ionic strength, and temperature. Moreover, they were not cytotoxic against A375, MCF7, and HeLa carcinogenic cell lines, as well as against fibroblast healthy cells, and their luminescence was maintained after cell internalization of the particles, allowing intracellular localization by flow cytometry and fluorescence confocal microscopy. The particles are, thus, excellent candidates for bioimaging.

## Figures and Tables

**Table 1:** Chemical composition determined by ICP and TGA analyses of most matured Tb^3+^:cit-cAp samples prepared by varying *x* from 0.001 to 0.020 M Tb^3+^.

*x*, time mol/L Tb^3+^	Ca (wt%)	P (wt%)	Tb (wt%)	(Ca + Tb)/P (mol)	H_2_O_ads_ (wt%)	H_2_O_str/b_ (wt%)	cit (wt%)	CO_3_^2−^ (wt%)
0.001 M, 96 h	31.8 ± 0.7	15.6 ± 0.4	1.59 ± 0.04	1.60 ± 0.01	3.2 ± 0.3	1.8 ± 0.2	1.9 ± 0.2	3.2 ± 0.3
0.001 M, 7 d	31.1 ± 0.2	15.2 ± 0.2	1.50 ± 0.01	1.60 ± 0.01	3.4 ± 0.3	2.0 ± 0.2	1.7 ± 0.2	3.5 ± 0.3
0.005 M, 96 h	26.8 ± 0.7	14.2 ± 0.4	7.33 ± 0.13	1.55 ± 0.01	3.5 ± 0.3	2.0 ± 0.2	1.3 ± 0.1	3.7 ± 0.4
0.005 M, 7 d	25.9 ± 0.4	14.0 ± 0.2	7.51 ± 0.11	1.53 ± 0.01	3.9 ± 0.4	2.1 ± 0.2	1.3 ± 0.1	3.7 ± 0.4
0.010 M, 96 h	21.5 ± 0.4	12.7 ± 0.2	12.80 ± 0.18	1.51 ± 0.01	6.0 ± 0.6	2.7 ± 0.3	1.8 ± 0.2	4.4 ± 0.4
0.010 M, 7 d	22.2 ± 0.2	13.7 ± 0.1	14.20 ± 0.16	1.45 ± 0.01	4.5 ± 0.4	2.7 ± 0.3	1.5 ± 0.1	3.6 ± 0.4
0.015 M, 7 d	17.0 ± 0.6	11.8 ± 0.4	18.72 ± 0.73	1.43 ± 0.01	7.6 ± 0.8 *	3.2 ± 0.3 *	2.2 ± 0.2 *	2.6 ± 0.3 *
0.020 M, 7 d	14.5 ± 0.4	11.2 ± 0.4	21.93 ± 0.82	1.39 ± 0.01	10.9 ± 1.1 *	4.4 ± 0.4 *	2.6 ± 0.3 *	2.3 ± 0.2 *

* These values are affected by the presence of both amorphous TbPO_4_·*n*H_2_O and TbPO_4_·H_2_O (rhabdophane) particles; H_2_O_ads_ is adsorbed water; H_2_O_str/b_ is structural and tightly bound water.

## Data Availability

Not applicable.

## Supplementary Materials

The following supporting information can be downloaded at: https://www.mdpi.com/article/10.3390/nano12081257/s1, Figure S1: XRD diagrams of samples precipitated at 4, 24, 96 and 7 days at Tb^3+^doping concentrations: (a) *x* = 0,015 M; (b) *x* = 0.02 M. *****(Apatite phase, PDF 01-1008). ^Δ^(TbPO_4_·*n*H_2_O phase, PDF 20-1244); Figure S2: TGA curves of samples prepared at (a) 96 h or (b) 7 days of maturation. (c) Representative TGA with DTG curve of 0.005 M Tb^3+^ sample with interpretation of mass losses; Figure S3: FESEM micrographs of samples precipitated at 7 days at Tb^3+^doping concentrations: (a,d) *x* = 0.005 M; (b,e) *x* = 0.010 M, and (c,f) 0.015 M; Figure S4: FTIR diagrams of samples precipitated at 4 h, 24 h, 96 h and 7 days, at different Tb^3+^doping concentrations: (a) *x* = 0; (b) *x* = 0.001 M; (c) *x* = 0.005 M, (d) *x* = 0.010 M; (e) *x* = 0.015 M, and (f) *x* = 0.020 M; Figure S5: Evolution of Raman spectra with maturation times of samples prepared with different Tb^3+^ doping concentrations: (a) *x* = 0; (b) *x* = 0.001 M; (c) *x* = 0.005 M and (d) *x* = 0.010 M; (e) *x* = 0.015 M, and (f) *x* = 0.02 M; Figure S6: Excitation (dashed lines) and emission (solid lines) uncorrected spectra of samples prepared with *x* = 0.02 M Tb^3+^ at maturation times of 96 h using *t_d_* = 120 µs, *t_d_* = 5 ms and (a) *λ_exc/em_* = 230/545 nm, slit width_exc/em_ = 2.5/2.5 nm, detector voltage 545 V; (b) *λ_exc/em_* = 375/545 nm, slit width_exc/em_ = 5/5 nm, detector voltage 470 V. Figure S7: Excitation (dashed lines) and emission (solid lines) uncorrected spectra of solid Tb^3+^: cit-cAp samples prepared with different Tb^3+^ doping concentration at maturation times of 4 h, 24 h, 96 h and 7 days; Figure S8:Variation of the R.L.I. of the Tb^3+^:cit-cAp samples at the maximum excitation and emission wavelengths prepared at several Tb^3+^ doping concentrations when the maturation time is changed. Figure S9: Variation of the R.L.I. of the Tb^3+^:cit-cAp samples at the maximum excitation and emission wavelengths at several maturation time when the Tb^3+^ doping concentration is changed; Figure S10: Luminescence decay curve of different Tb^3+^:cit-cAp samples at maturation times of 96 h, *t_d_* = 100 µs, *t_g_* = 0.01 ms, *λ_exc/em_* = 375/545 nm, slit-widths_exc/em_ = 10/10 nm, and detector voltage = 600 V. Circles correspond to experimental data (100 cycles) and lines to the fitting equation; Figure S11: Variation of the luminescence lifetime of the Tb^3+^:cit-cAp samples prepared at several Tb^3+^ doping concentrations when the maturation time is changed; Figure S12: Variation of the luminescence lifetime of the Tb^3+^:cit-cAp samples at several maturation times when the Tb^3+^ doping concentration is changed. Figure S13: Effect of the ionic strength over the (a) R.L.I. and (b) luminescence lifetime of the Tb^3+^:cit-cAp samples prepared with different Tb^3+^ doping concentrations at 96 h maturation time dispersed in aqueous media. Figure S14. Effect of the temperature over the (a) R.L.I. and (b) luminescence lifetime of the Tb^3+^: cit-cAp samples prepared with different Tb^3+^ doping concentrations at 96 h maturation time dispersed in aqueous media.
