# Supplementary material for: Luminescent Citrate-Functionalized Terbium-Substituted Carbonated Apatite Nanomaterials: Structural Aspects, Sensitized Luminescence, Cytocompatibility, and Cell Uptake Imaging
Source: Nanomaterials (Basel). 2022 Apr 7;12(8):1257. doi: 10.3390/nano12081257 (PMC9032902; doi:10.3390/nano12081257)
Supplement: Supplementary file 1 [file nanomaterials-12-01257-s001.zip › nanomaterials-1645347-supplementary.pdf]

*Supplementary Materials*

# Luminescent Citrate-Functionalized Terbium-Substituted Carbonated Apatite Nanomaterials: Structural Aspects, Sensitized Luminescence, Cytocompatibility, and Cell Uptake Imaging

**Jaime Gómez-Morales [1,*], Raquel Fernández-Penas [1], Francisco Javier Acebedo-Martínez [1], Ismael Romero-Castillo [1], Cristóbal Verdugo-Escamilla [1], Duane Choquesillo-Lazarte [1], Lorenzo Degli Esposti [2], Yaiza Jiménez-Martínez [3], Jorge Fernando Fernández-Sánchez [4], Michele Iafisco [2] and Houria Boulaiz [3]**

[1] Laboratorio de Estudios Cristalográficos. IACT-CSIC-UGR. Avda. Las Palmeras, n° 4. 18100 Armilla, Spain; raquel@lec.csic.es (R.F.-P.); j.acebedo@csic.es (F.J.A.-M.); ismaelrc92@gmail.com (I.R.-C.); cristobal.verdugo@csic.es (C.V.-E.); duane.choquesillo@csic.es (D.C.-L.)

[2] Institute of Science and Technology for Ceramics (ISTEC), National Research Council (CNR), Via Granarolo 64, 48018 Faenza, Italy; lorenzo.degliesposti@istec.cnr.it (L.D.E.); michele.iafisco@istec.cnr.it (M.I.)

[3] Instituto de Biopatología y Medicina Regenerativa (IBIMER), Universidad de Granada, 18016 Granada, Spain; yaijmartinez@correo.ugr.es (Y.J.-M.); hboulaiz@ugr.es (H.B.)

[4] Department of Analytical Chemistry, Faculty of Sciences, University of Granada, Avda. Fuentenueva s/n, 18071 Granada, Spain; jffernan@ugr.es

* Correspondence: jaime@lec.csic.es; Tel.:+34-958525020 or +34-958230000 (ext. 436640)

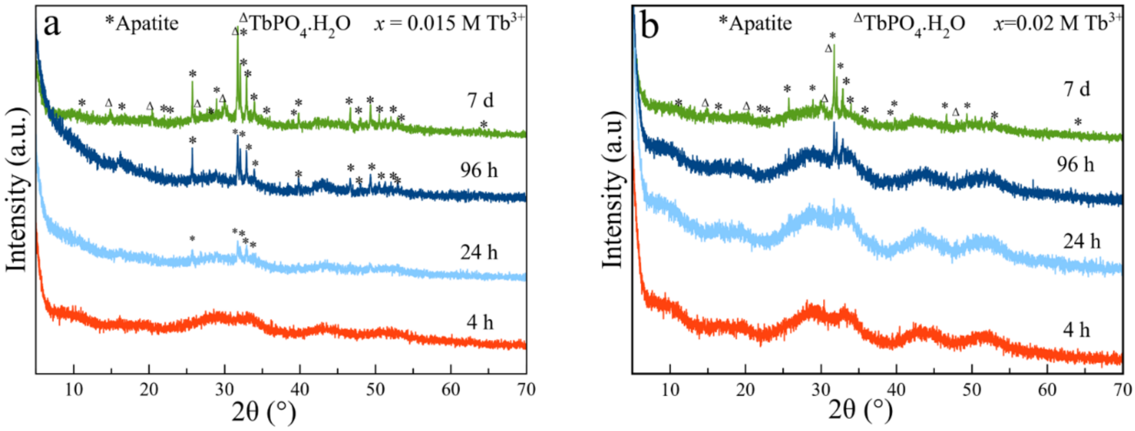


**Figure S1.** XRD diagrams of samples precipitated at 4, 24, 96 and 7 days at $Tb^{3+}$doping concentrations: (a) $x$ = 0.015 M; (b) $x$ = 0.020 M. *(Apatite phase, PDF 01-1008). $^{\Delta}$($TbPO_4.H_2O$ phase, PDF 20-1244).

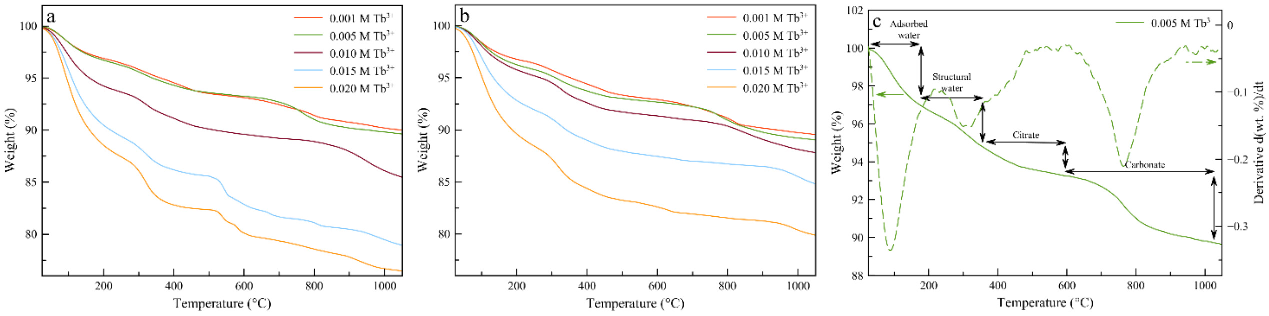


**Figure S2.** TGA curves of samples prepared at (a) 96 h or (b) 7 days of maturation. (c) Representative TGA with DTG curve of 0.005 M $Tb^{3+}$ sample with interpretation of mass losses.

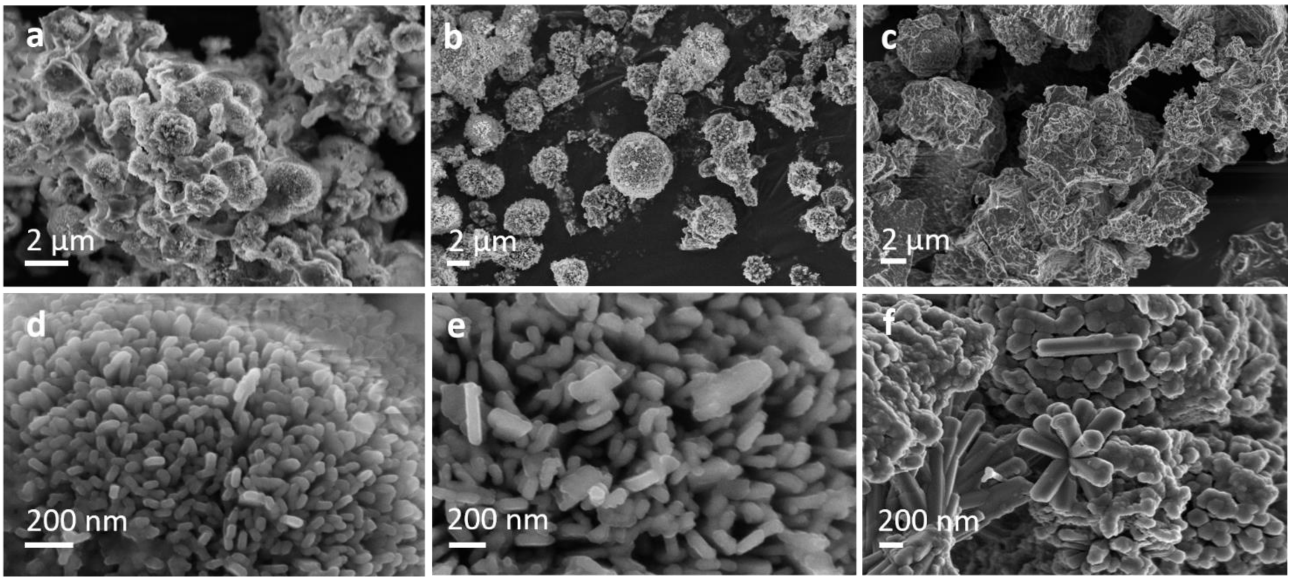


**Figure S3.** FESEM micrographs of samples at 7 days of maturation at $Tb^{3+}$ doping concentration of: (a,d) $x$ = 0,005 M; (b,e) $x$ = 0.010 M, and (c,f) 0.015 M

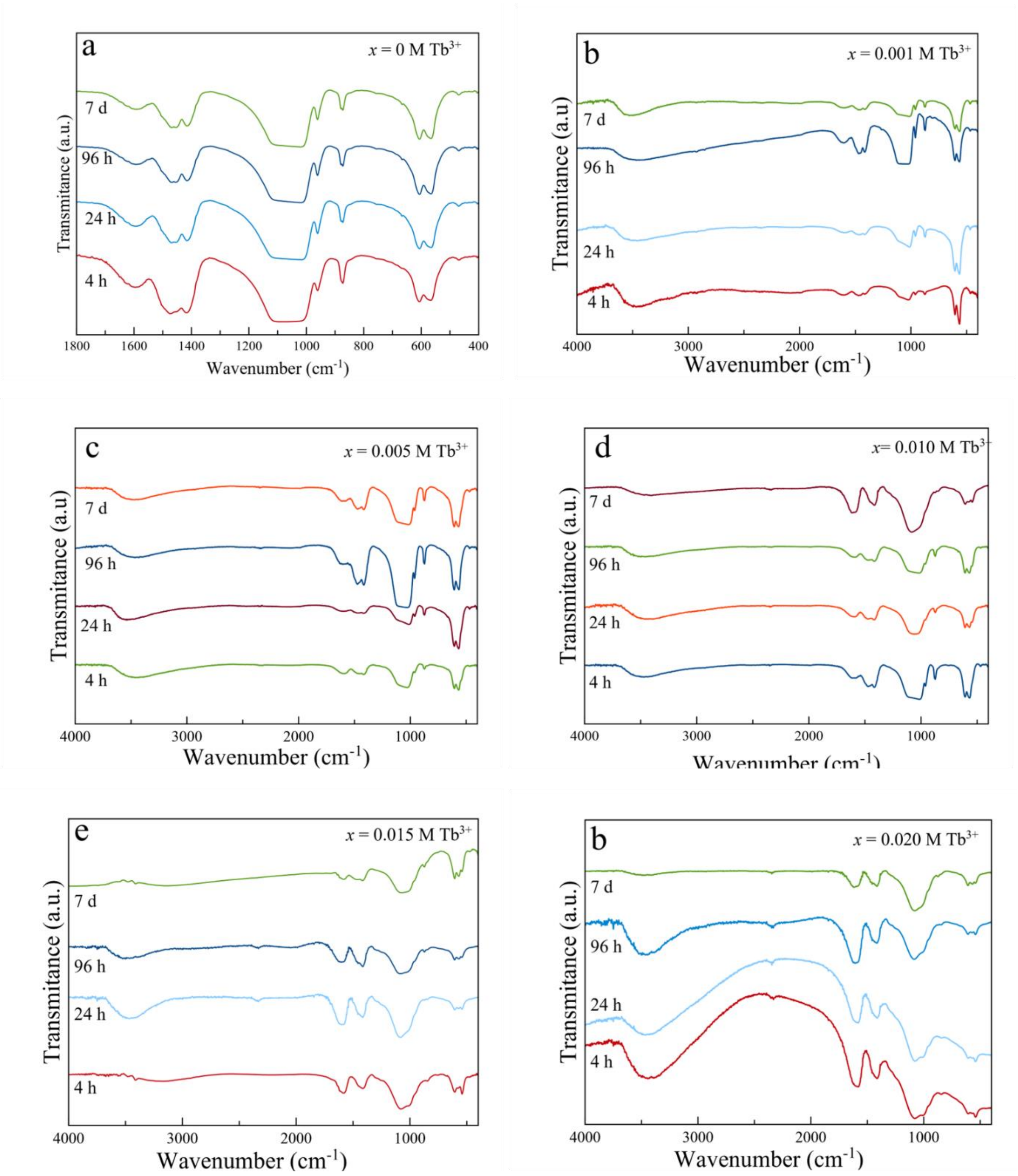


**Figure S4**. FTIR spectra of samples precipitated at 4 h, 24 h, 96 h and 7 days at $Tb^{3+}$ doping concentrations of (a) $x$ = 0; (b) $x$ = 0.001 M; (c) $x$ = 0.005 M, (d) $x$ = 0.010 M; (e) $x$ = 0.015 M, and (f) $x$ = 0.020 M.

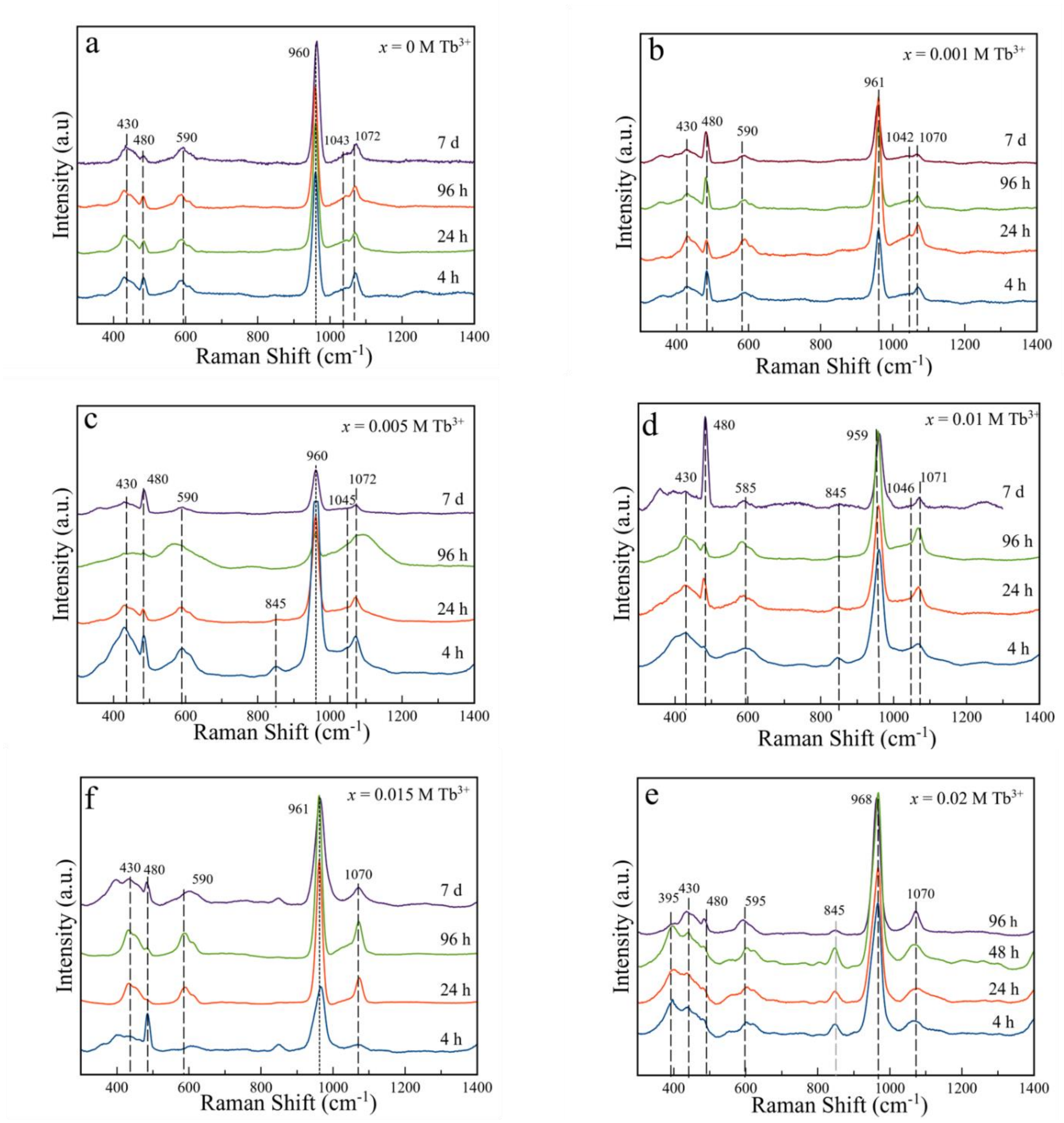


**Figure S5.** Evolution of Raman spectra in function of maturation time of samples with $Tb^{3+}$ doping concentrations of (a) $x$ = 0; (b) $x$ = 0.001 M; (c) $x$ = 0.005 M and (d) $x$ = 0.010 M; (e) $x$ = 0.015 M, and (f) $x$ = 0.02 M.

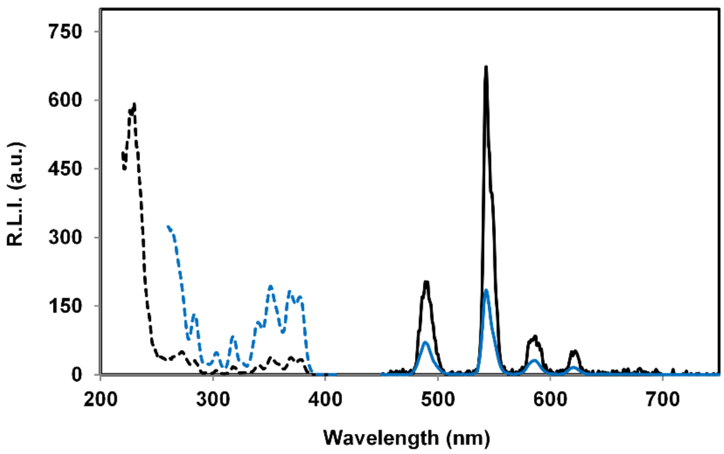


**Figure S6.** Uncorrected excitation (dashed lines) and emission (solid lines) spectra of samples prepared with $x$ = 0.02 M $Tb^{3+}$ at maturation time of 96 h using $t_d$ = 120 µs, $t_d$ = 5ms and a) $\lambda_{exc/em}$ = 230/545 nm, slit width$_{exc/em}$ =2.5/2.5 nm, detector voltage 545 v; (b) $\lambda_{exc/em}$ =375/545 nm, slit width$_{exc/em}$ =5/5 nm, detector voltage 470 v.

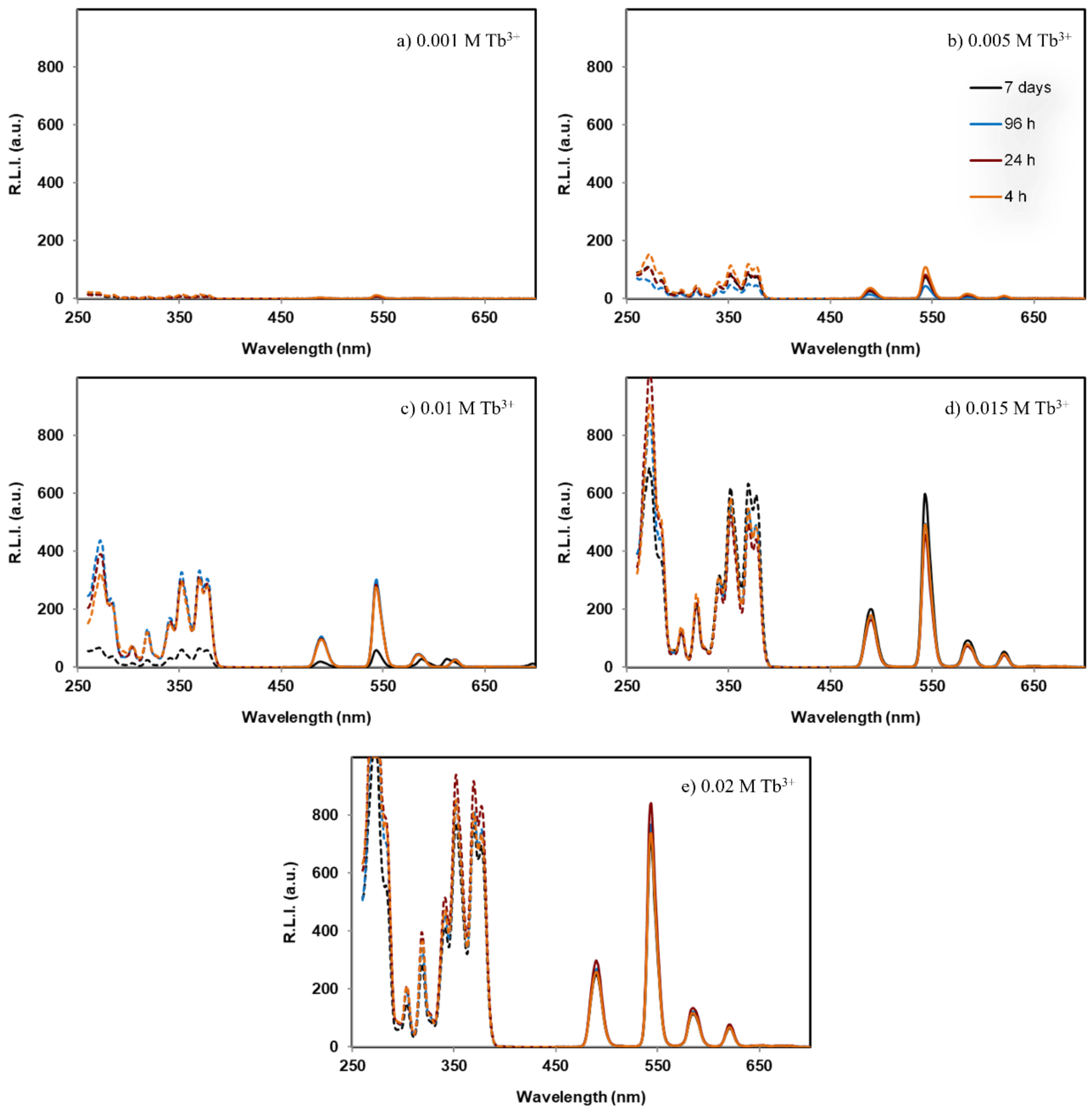


**Figure S7**. Uncorrected excitation (dashed lines) and emission (solid lines) spectra of solid $Tb^{3+}$: cit-cAp samples prepared with different $Tb^{3+}$ doping concentration at maturation times of 4h, 24h, 96 h and 7 days.

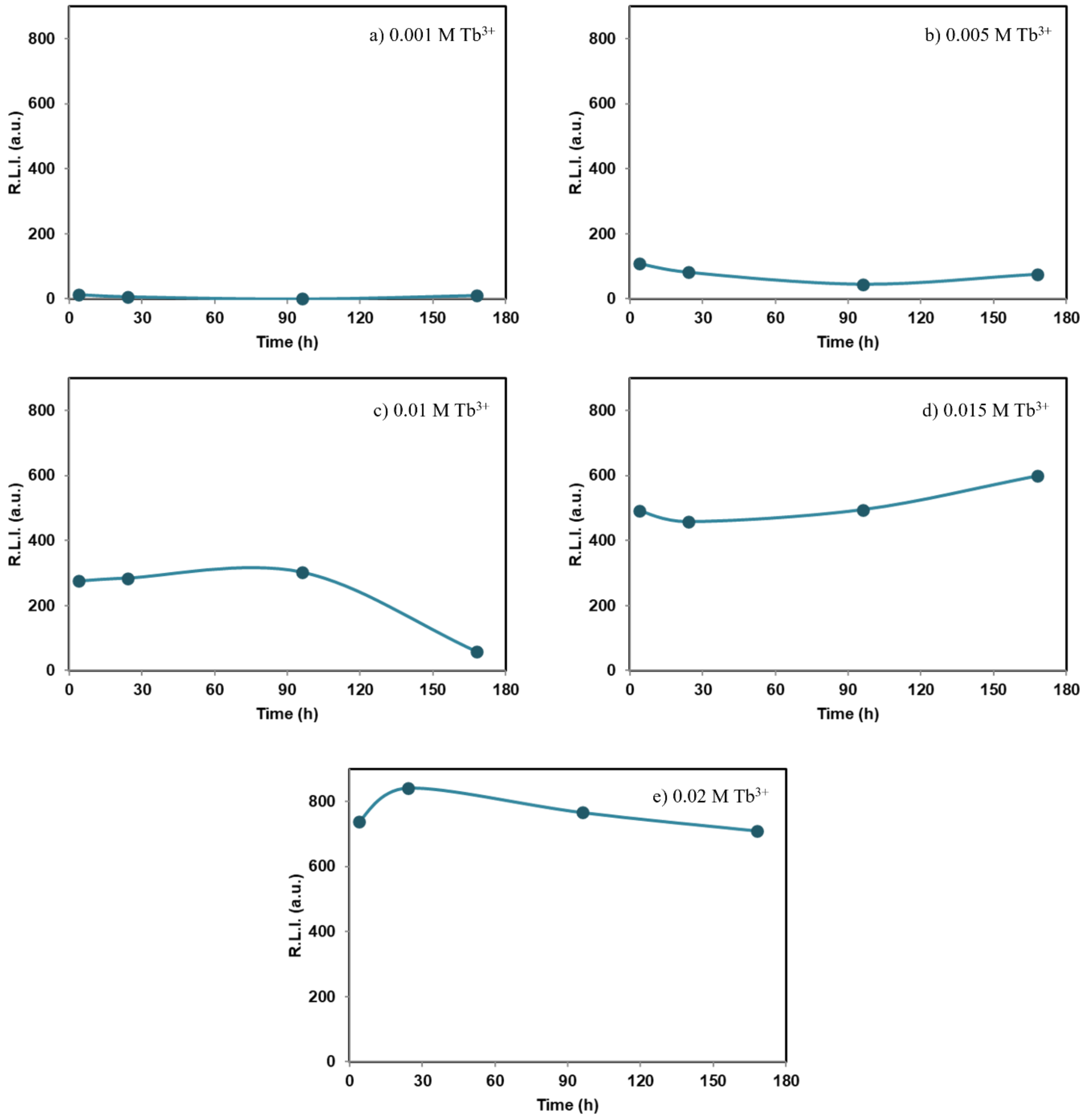


**Figure S8**. Variation of the R.L.I. in function of maturation time of the $Tb^{3+}$:cit-cAp samples at the maximum excitation and emission wavelengths prepared at several $Tb^{3+}$ doping concentrations.

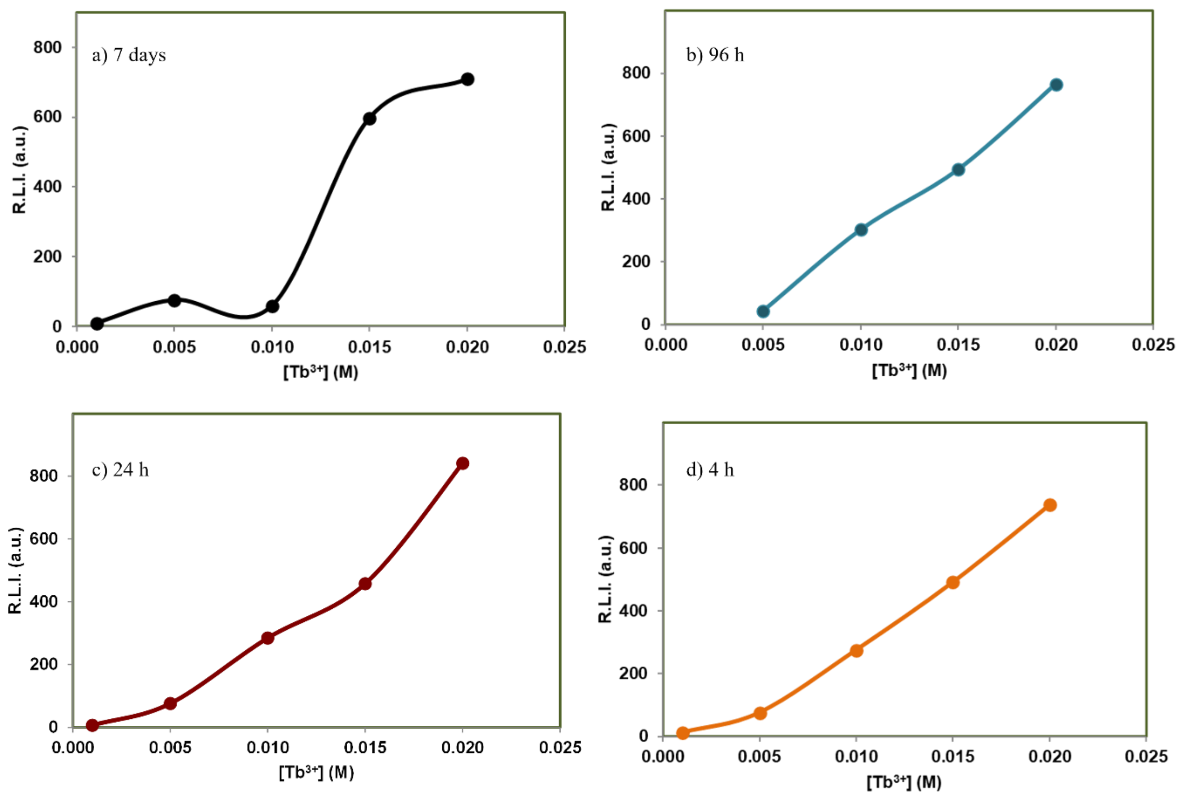


**Figure S9.** Variation of the R.L.I. in function of $Tb^{3+}$ doping concentration of the $Tb^{3+}$:cit-cAp samples at the maximum excitation and emission wavelengths at several maturation times.

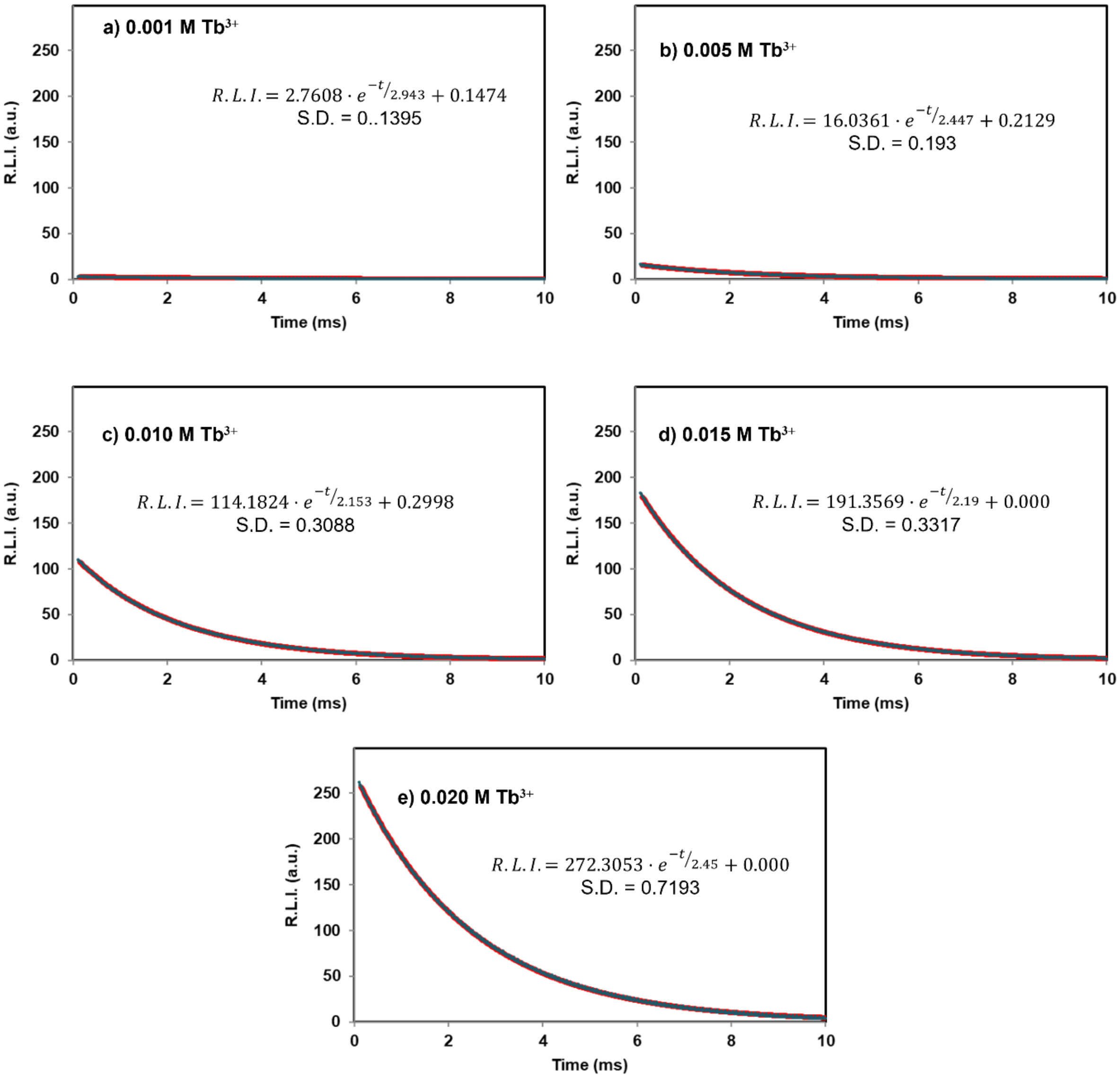


**Figure S10**. Luminescence decay curve of different $Tb^{3+}$:cit-cAp samples at maturation times of 96h, $t_d$ = 100 µs, $t_g$ = 0.01ms, $\lambda_{exc/em}$ = 375/545 nm, slit-widths$_{exc/em}$ = 10/10 nm, and detector voltage = 600 V. Circles correspond to experimental data (100 cycles) and lines to the fitting equation.

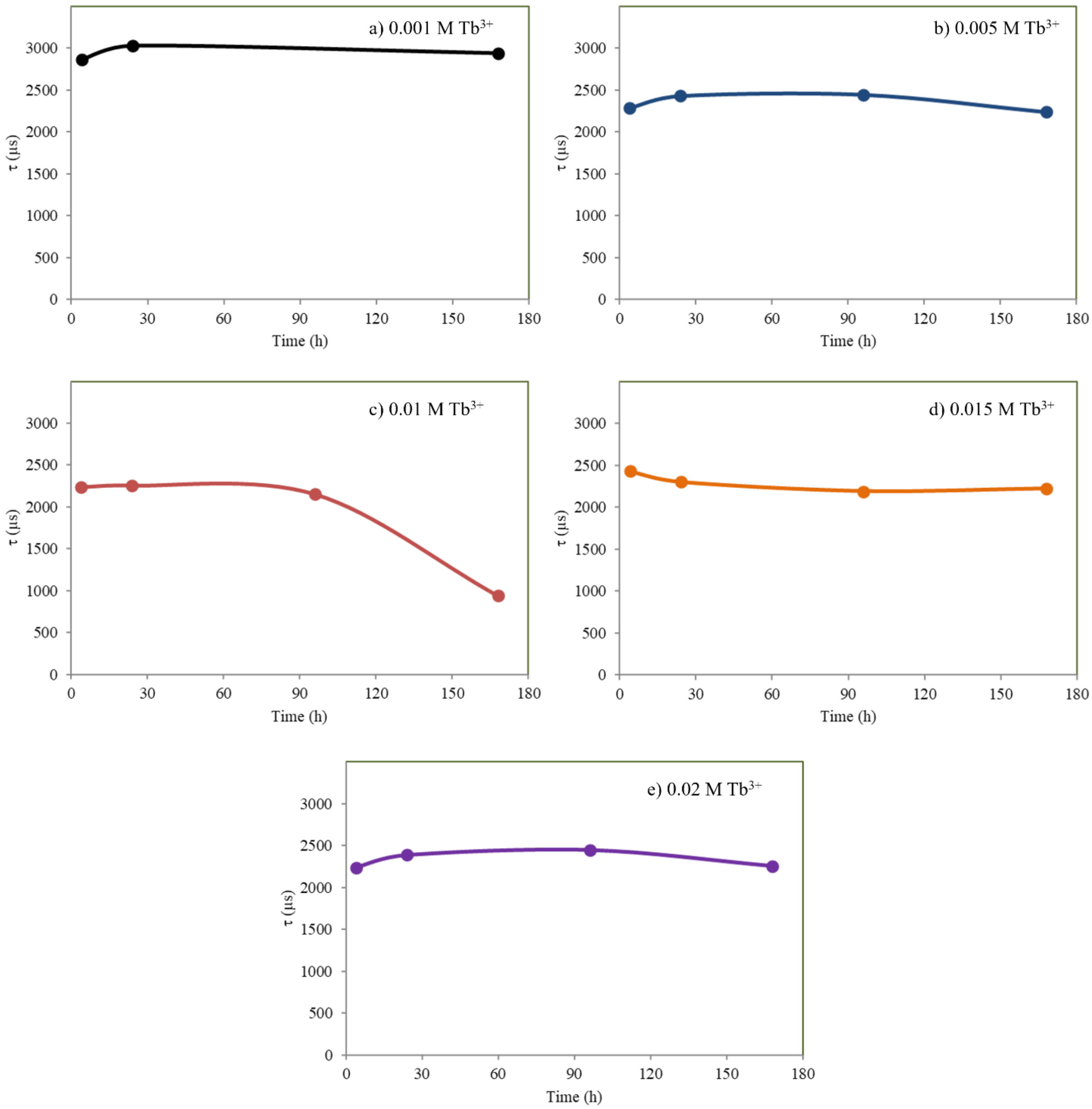


**Figure S11**. Variation of the luminescence lifetime in function of maturation time of the $Tb^{3+}$:cit-cAp samples prepared at several $Tb^{3+}$ doping concentrations.

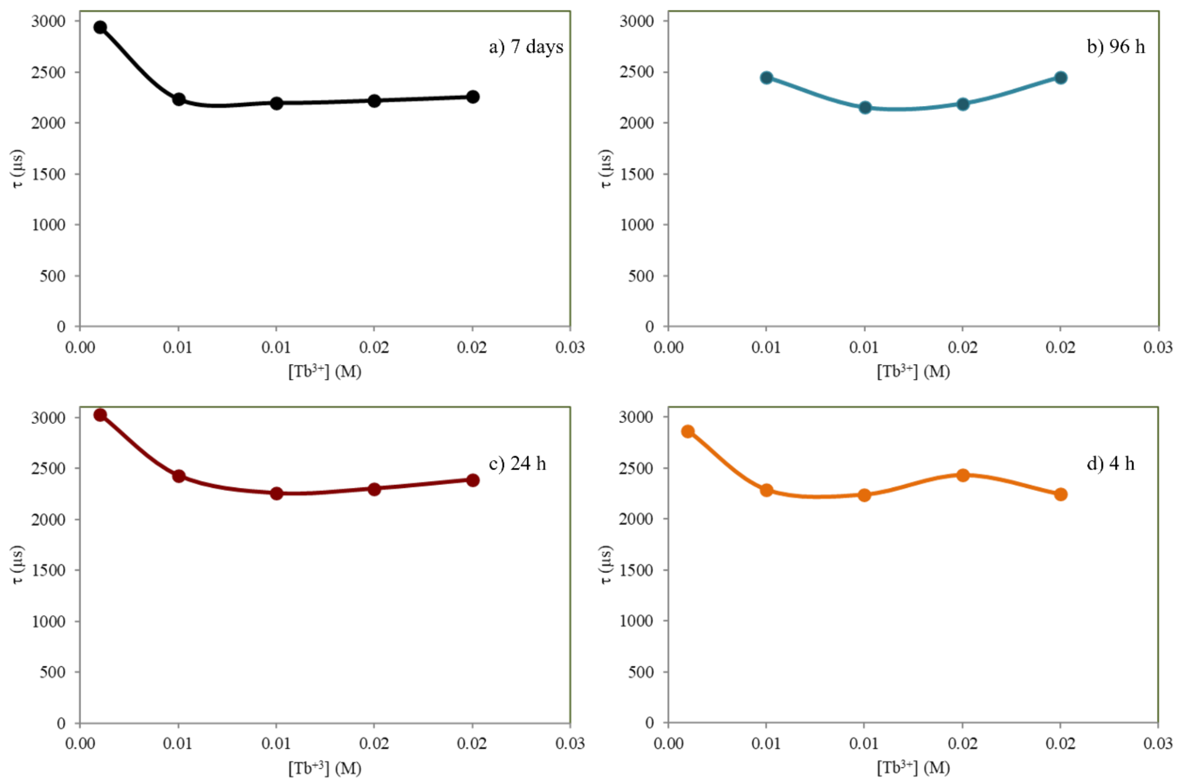


**Figure S12**. Variation of the luminescence lifetime in function of $Tb^{3+}$ doping concentration of the $Tb^{3+}$:cit-cAp samples at several maturation times.

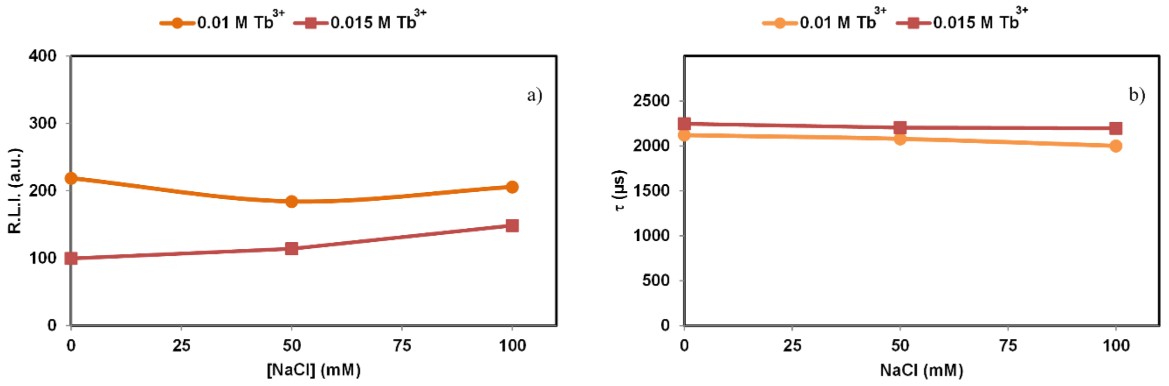


**Figure S13**. Effect of the ionic strength over the (a) R.L.I. and (b) luminescence lifetime of the $Tb^{3+}$:cit-cAp samples prepared with different $Tb^{3+}$ doping concentrations at 96 h maturation time dispersed in aqueous media.

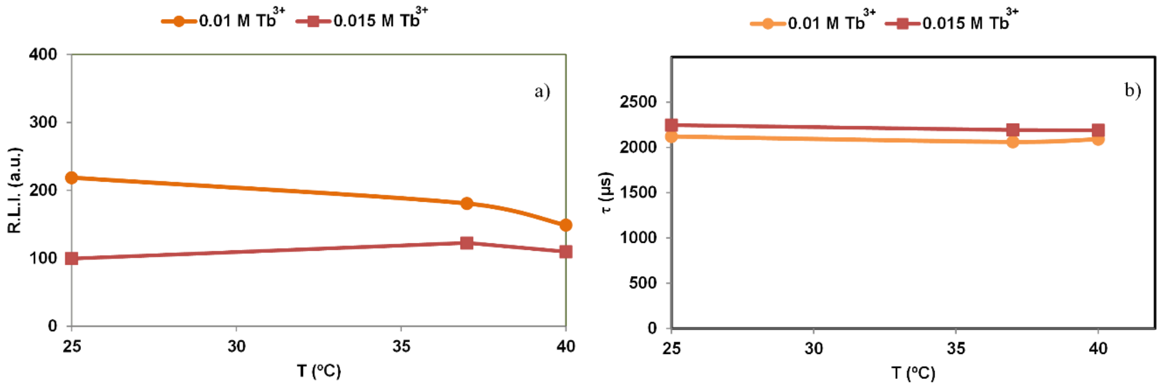


**Figure S14.** Effect of the temperature over the (a) R.L.I. and (b) luminescence lifetime of the $Tb^{3+}$:cit-cAp samples prepared with different $Tb^{3+}$ doping concentrations at 96 h maturation time dispersed in aqueous media.
